# Insight into the Functional Dynamics and Challenges of Exosomes in Pharmaceutical Innovation and Precision Medicine

**DOI:** 10.3390/pharmaceutics16060709

**Published:** 2024-05-24

**Authors:** Anu Sharma, Anita Yadav, Aparajita Nandy, Subhadip Ghatak

**Affiliations:** McGowan Institute for Regenerative Medicine, Department of Surgery, University of Pittsburgh, Pittsburgh, PA 15219, USA; ans831@pitt.edu (A.S.); any102@pitt.edu (A.Y.); apn44@pitt.edu (A.N.)

**Keywords:** exosome therapy, regenerative medicine, EV-based therapeutics regulatory affairs, cell–cell crosstalk, FDA, precision medicine

## Abstract

Of all the numerous nanosized extracellular vesicles released by a cell, the endosomal-originated exosomes are increasingly recognized as potential therapeutics, owing to their inherent stability, low immunogenicity, and targeted delivery capabilities. This review critically evaluates the transformative potential of exosome-based modalities across pharmaceutical and precision medicine landscapes. Because of their precise targeted biomolecular cargo delivery, exosomes are posited as ideal candidates in drug delivery, enhancing regenerative medicine strategies, and advancing diagnostic technologies. Despite the significant market growth projections of exosome therapy, its utilization is encumbered by substantial scientific and regulatory challenges. These include the lack of universally accepted protocols for exosome isolation and the complexities associated with navigating the regulatory environment, particularly the guidelines set forth by the U.S. Food and Drug Administration (FDA). This review presents a comprehensive overview of current research trajectories aimed at addressing these impediments and discusses prospective advancements that could substantiate the clinical translation of exosomal therapies. By providing a comprehensive analysis of both the capabilities and hurdles inherent to exosome therapeutic applications, this article aims to inform and direct future research paradigms, thereby fostering the integration of exosomal systems into mainstream clinical practice.

## 1. Introduction

The expanding domain of exosome research represents a significant frontier in the realm of pharmaceutical innovation and precision medicine, offering a convergence of novel opportunities and complex challenges [1,2]. Exosomes, defined as nanosized extracellular vesicles (EVs) of endosomal origin, secreted by almost all cell types, have gained prominence as potential vectors for therapeutic delivery [3,4]. Their intrinsic characteristics, such as biological stability, minimal immunogenicity, and the capability for cell-specific targeting, position them as pivotal candidates in the advancement of drug delivery systems, nucleic acid, and vaccine therapeutics [5,6]. In this review, we have used the term “exosomes” with the intent of discussing the small EVs of endosomal origin having diameters ranging from 30 to 150 nm. It is critically important to acknowledge the fact that the terms “exosome” and “small EV” are not synonymous and should be used with precision. This review seeks to dissect the multifaceted landscape of exosome therapy and show how it is more advantageous than liposomal and microvesicle-based therapies (Table 1) within the pharmaceutical innovation sphere and precision medicine, by focusing on the crucial aspects of regulatory scrutiny, particularly by the United States Food and Drug Administration (FDA). It also aims to analyze the hurdles intrinsic to translating exosome-based therapies from benchtop research to bedside clinical practices [7,8]. The contemporary strategies devised to address the challenges endemic to the therapeutic application of exosomes encompass advancements in research methodologies, data standardization, and the precision characterization of these nanovesicles, alongside innovations aimed at enhancing targeting specificity and cargo delivery efficiency [9]. It is time to critically explore the emerging techniques for single-exosome analysis and the refinement of surface modification approaches [10,11]. Such advancements underline the transition of exosomes from conceptual entities to practical therapeutic vectors, highlighting the importance of biochemical engineering in optimizing their clinical applicability [12]. By navigating through this discourse, we aim to trace the developmental trajectory of exosome-based therapeutic applications, emphasizing the dynamic synergy between scientific research and regulatory governance, as well as the commitment to ensuring patient safety and therapeutic efficacy [13]. This study not only seeks to contribute to the scholarly discourse on exosome therapy but also aspires to catalyze future advancements in therapeutic modalities, ushering in a new epoch of patient-focused care and therapeutic delivery.

## 2. Where Does the FDA Stand?

**Figure 1:**
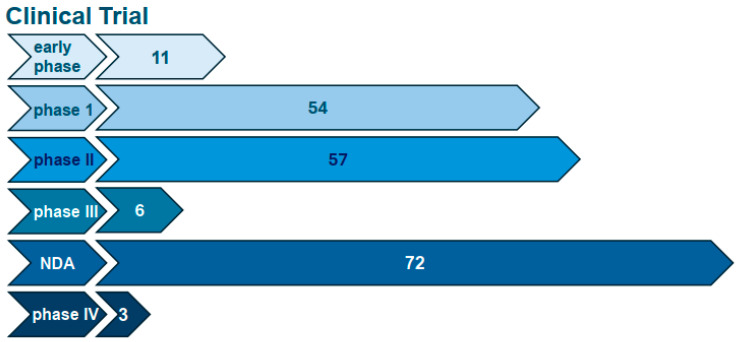
Exosome-based therapy in clinical trials (as listed on clinicaltrials.gov as accessed on 20 April 2024).

Within the broader domain of regenerative medicine products, the U.S. FDA categorizes therapies derived from stem cells as somatic cellular therapies. It is important to note that the FDA employs a tailored regulatory framework for stem cell-based products based on their characteristics and intended uses. Recent work by Han et al. highlighted the significant influence of paracrine factors such as exosomes on the therapeutic potential of stem cells [14]. The progression of therapeutic applications utilizing exosomes delineates an intricate balance between innovation in biomedical research and stringent oversight by regulatory entities, notably the US FDA [12]. The FDA’s comprehensive regulatory framework for extracellular vesicle-based therapies, particularly those derived from exosomes, is predicated on ensuring the transition of these modalities from theoretical constructs to practical clinical applications, adhering to the highest safety and efficacy standards [15,16]. Such a regulatory approach is predicated on the principles of scientific accuracy, robust quality control mechanisms, and adherence to regulatory compliance standards [17,18]. Exosomes, a subclass of EVs, are recognized for their promising therapeutic potential across various medical fields [19]. These vesicles provide several key advantages over conventional cell-based therapies by their inherent capability to circumvent the proliferative and differentiation-associated risks post-administration, unlike that of stem cells [20]. Furthermore, they offer the potential for enhanced consistency in quality control standards across different production batches, which is a critical consideration in the context of biological drug development [5]. However, the path to clinical application of these vesicles is fraught with complexities, necessitating a comprehensive elucidation of their biological mechanisms, the establishment of scalable production methodologies that ensure reproducibility, and a tangible enhancement of their intrinsic therapeutic potential [21]. A primary challenge in this developmental trajectory is the establishment of uniform manufacturing protocols that guarantee consistency across product batches. Given the classification of exosomes as biological drugs by the FDA [22,23], they are subjected to exhaustive evaluations to validate their safety and efficacy through extensive clinical trials [24]. This stringent evaluative process highlights the critical need for refined manufacturing processes, comprehensive human trials, and conclusive evidence demonstrating clinical benefits [25,26]. Figure 1 shows an analysis of exosome-based clinical trials, as listed on clinicaltrials.gov (20 April 2024). A search on clinicaltrials.gov using ‘exosome’, ‘exosome therapy’, and ‘exosome treatment’, as keywords generated 204 records and listed in Table 2.

To address the potential risks associated with unregulated exosome therapies, the FDA has proactively issued guidance and warnings concerning the marketing of unapproved exosome products [27]. These regulatory actions reflect the FDA’s dedication to safeguarding public health and maintaining the integrity of emerging therapeutic modalities. Through the enforcement of regulatory standards and the meticulous review of clinical trial submissions, the FDA endeavors to mitigate the risks posed by unauthorized exosome therapies. While the regulatory pathway for exosome-based therapies is devoid of specific guidance on delivery systems, applying the existing regulatory requirements for biological products remains imperative [27]. These include the submission of Investigational New Drug (IND) applications, adherence to Good Manufacturing Practice (GMP) protocols, implementation of quality control measures, and the selection of appropriate cell sources for therapeutic production [28].

## 3. Challenges and Prospects Associated with the Translation of Exosome Therapy

The clinical adoption of exosome-based therapeutic strategies is impeded by challenges associated with low exosomal yield and efficiency [7]. In a controlled laboratory setting, extraction from one milliliter of cell culture medium frequently results in less than one microgram of exosomal protein [29]. Strategies to augment exosome production have been explored, encompassing mechanical (e.g., three-dimensional culture systems and the application of shear stress), biochemical (e.g., treatment with lipopolysaccharide (LPS), interferon-gamma (IFN-γ), bone morphogenetic protein 2 (BMP-2), tumor necrosis factor-alpha (TNF-α), and hypoxia-inducible factor 1-alpha (HIF-1α)), and physical (e.g., thermal stress, hypoxic conditions, and nutrient deprivation) methodologies [30,31,32].

Exosome isolation is complicated by their intrinsic heterogeneity, manifested in variations in size, composition, surface markers, and biological origin [33]. Predominant isolation and purification methodologies are based on immunoaffinity, leveraging surface charge characteristics, or size and density differences [34]. Each methodology presents specific advantages and drawbacks, and no singular approach is universally applicable [35]. Ultracentrifugation, while often deemed the gold standard, is constrained in its scalability by factors such as cost, efficiency, vesicle aggregation, and the co-isolation of lipoproteins, despite minimal chemical and expertise requirements [36]. Conversely, immunoaffinity chromatography, exploiting specific antigen–antibody interactions, is noted for its high specificity, yield, and purity, albeit dependent on the presence of appropriate exosomal surface antigens [37]. Techniques predicated on size differentiation, including size-exclusion chromatography and ultrafiltration, are advantageous for scalability but are limited by issues such as pore clogging, loss of exosomal material, and reduced purity [38,39,40]. The amalgamation of disparate isolation techniques, incorporating precipitation- and microfluidics-based approaches, may offer a comprehensive solution to effectively address the multifaceted requirements for exosome isolation and purification [41]. For instance, in our research endeavors, a synergistic application of differential ultracentrifugation and immunomagnetic separation facilitated the isolation of cell-specific exosomes from murine tissue, and a parallel strategy was employed for the extraction of exosomes from human wound effluents [42,43].

Anticoagulants, notably EDTA, have been documented to markedly influence both the composition of exosomes and their isolation processes from blood specimens [44]. Subsequent investigations have elucidated that during the procedural phases of blood collection and processing, EDTA plays a stabilizing role in maintaining the integrity of platelet-derived EVs [45]. However, it has also been substantiated that the utilization of EDTA-containing tubes does not forestall alterations in the exosomal profile. The effects of EDTA substitutes on the characterization and profiles of exosomes remain underexplored, presenting a significant gap in current research [46]. This issue bears considerable importance for the reproducibility of quantifications of blood exosome concentrations and the isolation of platelet-derived exosomes, which pose considerable challenges in biomarker discovery for conditions not associated with platelets [47]. This aspect becomes critically pertinent when processing single-spun plasma samples, potentially leading to contaminations in the exosome isolates due to the presence of frozen platelets [48].

The selection of an anticoagulant that preserves the analytes of interest without compromising the integrity of the exosomes is of paramount consideration [47]. Reports indicate that blood collection tubes incorporating non-aldehyde-based stabilizers for cell-free nucleic acids are conducive for exosome handling, as they also contribute to exosome stabilization. [49]. Another essential aspect for consistent determination of exosome composition in blood involves understanding how various pre-analytical factors, such as collection, processing, and storage conditions, impact the protein corona and the co-isolation of exosomes with other blood components, such as platelets, lipoproteins, soluble protein aggregates, viruses, cell-free DNA/histones, or circulating mitochondria [50].

Furthermore, the quantification and characterization of blood-derived exosomes are also contingent upon the centrifugation parameters and storage conditions [51]. The integrity of microRNA analyses in exosome samples can be significantly compromised by hemolysis during blood sample collection [52]. Addressing these variables is crucial in the standardization of methodologies for exosome research.

## 4. Factors Taken into Consideration for Exosomal Function Dictation

The function of exosomes is determined by isolation techniques, molecular characterization, functional tests, and imaging approaches. Despite progress, challenges remain due to the need for standardization, as well as the limitations of current technology [53]. Understanding the methods of exosome uptake, such as endocytosis, membrane fusion, or receptor-mediated interactions, is essential for the successful delivery of functional biomolecular cargo such as proteins, RNA, or miRNA [54]. Advanced in vitro assays such as fluorescent labeling and reporter gene assays, in vivo models, molecular analysis, such as proteomics, transcriptomics, Western blotting, qPCR, functional impact studies, such as cell proliferation assays, apoptosis assays, migration and invasion assays, and immunomodulation assays, help elucidate the functional impacts of exosomes on recipient cells [55]. The issues of cell specificity, dose, safety, and scalability must be addressed to maximize therapeutic efficacy, while minimizing unwanted effects. Exosomes have intrinsic targeting capability influencing both physiological and pathological states through lipids, RNA and proteins. Not surprisingly, various biotechnology companies like Stem Cell Medicine Ltd., Evox Therapeutics, Pharmaceutics Inc. are working to create different therapies using exosomes [55]. Much research has been conducted to elucidate the functioning of exosomes following uptake into recipient cells, overriding their inherent targeting ability. Exosomes can enter a recipient cell by macropinocytosis, phagocytosis, lipid-raft-mediated uptake, and membrane fusion. The primary route of exosome uptake is clathrin/caveolin-mediated endocytosis [54,56,57]. Functional delivery requires exosome-encapsulated material to “escape” from the endosome and enter the cytoplasm [56]. To support the exosome-encapsulated content’s communicative qualities, robust functional transport to recipient cells must be shown. Endosomal escape and effective cargo delivery are desired outcomes for exosome therapeutic usage, but there is also a risk of cargo loss due to breakdown, recycling in the cell, or the re-release of intact vesicles into the extracellular environment [56].

### 4.1. Protein Corona

**Figure 2:**
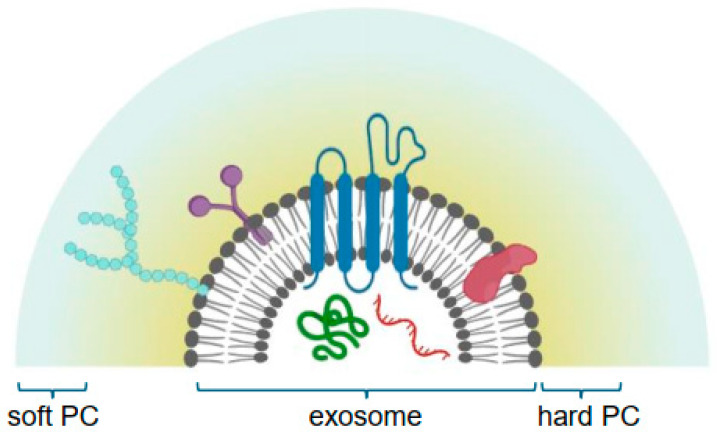
Illustration of the protein corona formation around exosomes.

The concept of the protein corona refers to the phenomenon where proteins adhere to the surface of exosomes upon their introduction into biological fluids such as blood plasma, forming a coating (Figure 2) [58]. This process alters the surface properties, such as shape, size, and structural modifications of exosomes, significantly affecting their interactions with the cell membrane of recipient cells by modifying recognition, uptake, targeting, and the biological responses elicited, with profound implications for health and diseases like cancer, Alzheimer’s, wound healing, etc. [59]. The protein corona comprises not merely a random assortment of plasma proteins, but includes specific proteins, such as anti-thrombin III, factor V, complement C3, IgG, fibronectin, and complement factor H, which influence the biological behavior and interactions of exosomes within the bloodstream [60]. This aspect facilitates the targeted delivery of exosomes to specific cell types or tissues by promoting interactions with the receptors. Nonetheless, the presence of a protein corona can compromise therapeutic efficacy through mechanisms such as the accelerated clearance of exosomes, induction of adverse immunological responses or reduction in the efficiency of cargo delivery to target cells [61].

Strategies involving engineering the exosome surface with specific ligands or coatings can alter the protein corona composition, enhancing targeting specificity and reducing unwanted immune reactions [62]. Pre-exposing exosomes to plasma or specific proteins, such as anti-thrombin III, factor V, complement C3, IgG, fibronectin, and complement factor H, before therapeutic applications, such as cancer, nervous system diseases, and immune diseases, can induce a favorable corona formation, potentially augmenting their therapeutic efficacy in vivo. Wolfram et al. highlighted the role of nanoparticles high surface free energy in the adsorption of molecules, predominantly proteins, leading to the formation of the protein corona [61]. The binding forces facilitating such interactions encompass Van der Waals forces, hydrophobic interactions, hydrogen bonds, electrostatic attractions, and π-π stacking [61].

Conceptually, the protein corona is divided into a ‘hard’ and a ‘soft’ layer, distinguished by the proximity of biomolecules such as proteins, lipids, and sugars to the nanovesicles [63]. The soft layer is characterized by a more dynamic exchange of biomolecules. However, there is debate regarding the heterogeneity of the hard corona formed in human blood plasma, with some sources indicating fewer than 100 distinct proteins, while others suggest greater than 100 [64]. Table 3 lists protein coronas adsorbed around exosomes [58].

The composition of the protein corona is influenced by the size, shape, and surface charge of the nanovesicles, with prevalent proteins including albumin, complement proteins, apolipoprotein 1, and immunoglobulins [58]. Protein attachment to nanovesicles induces both reversible and irreversible structural changes in the proteins, occurring in stages, with the least stable conformations exhibiting the quickest misfolding kinetics [65]. Protein adsorption is enhanced, with an increase in size due to the lower curvature, facilitating more extensive surface interaction and consequent protein conformational changes. Additionally, a positive surface charge correlates with increased protein adsorption [61].

**Table 3:** List of protein coronas adsorbed around exosomes.

S. No.	Hard and Soft PC	Types of Interactions	Effect	References
1.	Albumin	Connects to RNA/DNA on exosomes	Phagocytosis of exosomes	Malonga et al. 2006 [66]
2.	ApoE and ApoB100	Protein–protein interactions	Increases transfer rate in peripheral tissue	Bertrand et al. 2017 [67]
3.	Apolipoprotein A1	Interacts with CD63 on Exosome surface	Phagocytosis of exosomes	Toth et al. 2021 [60]
4.	Apolipoprotein B	Interacts with CD63 on Exosome surface	Increases phagocytic activities and triggers secondary inflammation reactions	Toth et al. 2021 [60]
5.	Complement factors 3	Interacts with CD63 on Exosome surface	Increases phagocytic activities and triggers secondary inflammation reactions	Toth et al. 2021 [60]
6.	Complement proteins C3b and C3ib	Protein–protein interactions	Prolonged chronic inflammatory conditions	Conde et al. 2012 [68]
7.	Immunoglobulin heavy chains of (γ2 and γ4)	Protein–protein interactions	Reduces transit time and affects bio-distribution	Toth et al. 2021 [60]
8.	Mismatched MHC-I and II	On the surface of Exosomes	T-cell immune responses	Hiltbrunner et al. 2016 [69]
9.	S100-A8, LDL-receptor, CD14, HLA class I	Phosphatidylserine and tissue factor on Exosome surface	Dynamic activity	Buzas et al. 2018 [70]

The protein corona on exosomes can also influence the cellular uptake of nanovesicles by modulating their adhesion to the cell membrane, a process which can either inhibit or enhance cellular internalization [71]. Despite the consensus on the dynamic nature of the protein corona, specific binding events occur at the nano–plasma interface, and their sequential order remains underexplored. Recent studies indicate that the quantity of protein in the corona varies over time, although the types of proteins bound tend to remain stable [58,61]. Nevertheless, the variability in protein corona composition among extracellular vesicle populations presents challenges in controlling the protein corona via surface engineering strategies [61].

### 4.2. Interactions of Lipoproteins with Exosomes

The potential interactions between exosomes and lipoproteins have received minimal attention in research conducted over the last few decades [62]. Previous investigations have rigorously examined lipoproteins as exogenous impurities in EV samples. Notably, lipoproteins are identified at a minimum quintuple excess in comparison to exosomes in biological specimens processed for exosome isolation, posing a significant challenge due to their overlap in size with exosomes [72,73]. Both exosomes and lipoproteins are ubiquitously found in the bloodstream, sharing similar dimensions and densities. Plasma specimens comprise exosomes (40–120 nm), microvesicles (100 nm–1 μm), and apoptotic bodies (50 nm–2 μm), while lipoprotein subclasses, such as HDL (5–12 nm), LDL (18–25 nm), IDL (25–35 nm), VLDL (30–80 nm), chylomicron remnants (30–80 nm), and chylomicrons (75–1200 nm), exhibit size ranges overlapping with those of exosomes [62]. This congruence in size renders their separate isolation unfeasible [73,74,75]. Prior research has documented the presence of complexes formed between exosomes and lipoprotein-like structures in plasma from healthy human subjects [76]. Subsequent research corroborated these findings, observing exosome complexation with lipoproteins under physiological conditions [73,75,77]. Subsequent investigations, including the work of Lozano-Andres et al., utilizing cryogenic transmission electron microscopy, corroborated the association of EVs with lipoproteins and elucidated the consequential effects on EV detection and characterization [78]. Historically, the focus has been on treating lipoproteins as inert contaminants, neglecting the potential interactions between these two biological nanoparticles [39]. Proteomic analyses and immunogold transmission electron microscopy have indicated that lipoprotein components may engage in dynamic interactions with exosomes, leading to complex formation in both artificial and physiological milieus [79]. These complexes could influence exosome detection, characterization, cellular uptake, and subsequent biological effects. Techniques such as atomic force microscopy (AFM) or transmission electron microscopy (TEM) have been proposed to investigate the complexation phenomena between these. Collectively, these insights suggest that exosomes and lipoproteins can form complexes in various environments [80]. It is also paramount to acknowledge that exosome isolation techniques might foster non-physiological interactions between exosomes and lipoproteins [81]. Given these recent discoveries, it becomes imperative to explore the nature of these interactions and their potential mechanistic, therapeutic, and diagnostic ramifications at the exosome−lipoprotein interface [62].

### 4.3. Effects of Size, Morphology, and Surface Modification on Cellular Uptake of Exosomes

The physicochemical characteristics of nanovesicles significantly influence cellular internalization dynamics. It is pertinent to examine whether the methodologies employed for isolating enriched exosomal fractions impact their cellular uptake [82]. A comparative analysis of exosomes isolated via ultracentrifugation and polymer-based precipitation techniques demonstrated that the latter results in a narrower particle size distribution, accelerated absorption by target cells, and enhanced cellular motility [83]. Furthermore, differential efficiencies in exosome uptake were observed between vesicles of different cellular origins, highlighting the specificity of interaction between exosomes and target cells [2,84]. Endocytic pathways, including clathrin/caveolae-mediated endocytosis, phagocytosis, macropinocytosis, and pinocytosis, play critical roles in the size-dependent uptake of exosomes [84]. Smaller exosomes, typically ranging from 30 to 100 nm, are more likely internalized through clathrin-mediated endocytosis, while larger vesicles may prefer alternative pathways, such as caveolin-mediated endocytosis or macropinocytosis [85,86]. The preference for specific endocytic pathways based on size impacts the efficiency of uptake and subsequent intracellular routing, thereby affecting cargo delivery to targeted cellular compartments [87]. Additionally, the morphological characteristics of exosomes, such as their cup-shaped or spherical appearance under TEM, influence their binding affinity to cellular receptors and internalization rates [88]. Variations in preferred morphology are observed across different cell types [89]. The composition of the exosome membrane also affects its fusogenic capacity, facilitating direct cargo delivery into the cytoplasm by fusion with the cell membrane [90].

Exosomes present a diverse array of surface molecules, including proteins lipids, and glycans which can be exploited for targeted cellular interactions [91]. However, their targeting potential is sometimes limited. Innovative approaches such as surface functionalization can enhance targeting specificity [92]. For instance, Tian et al. enhanced the bioavailability of curcumin-loaded exosomes through peptide functionalization, enabling them to cross the blood-brain barrier post-intravenous injection [93]. Similarly, Liang et al. developed miR-140-loaded exosomes that, when administered intra-articularly, effectively penetrated dense extracellular matrices to alleviate osteoarthritis symptoms, suggesting a viable cell-free treatment strategy [94]. Moreover, Li et al. demonstrated the therapeutic potential of targeted engineered exosomes in diabetic wound healing [95]. Enhancements in the biofunctional engineering of exosomes could improve their targeting accuracy and circulation time, optimizing the delivery of therapeutic cargos to specific cells or tissues [95,96].

### 4.4. Cellular Environment

Paracrine intercellular communication via exosomes utilizes the cellular milieu as a conduit and modulates exosome–cell interactions to varying extents [97]. This milieu includes not only the extracellular matrix (ECM) but also external biophysical variables such as pH, temperature, and oxidative or hypoxic conditions [98]. For instance, ECM rigidity may influence exosome uptake, while its mechanical properties can dictate exosomal transit by interacting with water permeability [99]. Additionally, the extracellular environment’s pH and extracellular ion concentrations can significantly affect the stability, absorption, and release of exosomes [100]. Variations in ion levels might alter the composition and structural integrity of exosomal membranes, thereby modifying their interactions with recipient cells [101]. A low pH environment, commonly encountered in tumor settings, may enhance the malignancy of cancer cells by modulating exosome release and uptake [102].

Temperature also profoundly affects the cellular uptake of exosomes, with reduced temperatures markedly impeding this process, indicating its energy-dependent nature [103]. Pathological conditions such as cancer, inflammation, or infection led cells to alter their exosome secretion and composition [104]. For example, cancer cells typically secrete more exosomes containing oncogenic factors that facilitate tumor growth, angiogenesis, and metastatic site preparation [105].

Under conditions of oxidative stress or hypoxia, cells might emit exosomes laden with stress-response proteins or RNAs, influencing the survival and functionality of recipient cells [106]. The local tissue environment, which encompasses components of the ECM, cell–cell interactions, growth factors, and cytokines, also plays a crucial role in modulating exosome activity. Specific receptors within the tissue microenvironment can enhance the selectivity of exosome uptake by recipient cells [107]. Furthermore, oxidative stress can alter the protein content of exosomes from amnion-epithelial cells, promoting the release of inflammatory mediators that trigger inflammation [106]. Additionally, external factors such as ionizing radiation have been shown to enhance exosomal secretion pathways in breast cancer cells, as demonstrated by Jabbari et al., suggesting a potential mechanism for developing treatment resistance [108].

Exosomes play an important function in immunological regulation. They are released by antigen-presenting cells and can provide therapeutic advantages by suppressing or increasing the immune response [109,110]. Exosomes from antigen-presenting cells include major histocompatibility peptide complexes and costimulatory molecules that influence antigen-specific CD8^+^ and CD4^+^ responses. Antigen-specific T cells directly contact MHC–peptide complexes on exosomes, activating T cells. Exosomes produced by dendritic cells (DCs) can stimulate T and B cells and have been studied for their immunostimulatory characteristics in cancer treatment. Exosomes generated by dendritic cells (DCs) can activate T and B cells and have been investigated for immunostimulatory properties in cancer therapy [111,112]. They can increase immunological responses in vivo by transferring MHC–peptide complexes from DCs that have been exposed to an antigen to another DC that has not encountered the antigen [113].

### 4.5. Entry and Departure of Exosomes from the Circulation

Exosomes function as pivotal carriers of bioactive molecules, facilitating intercellular communication and playing a significant role in both paracrine and endocrine-signaling processes within the tissue interstitium and circulatory system [114,115]. Nevertheless, the precise biological mechanisms facilitating their ingress and egress from the circulatory system have yet to be fully elucidated. Prior research has delineated the traversal of exosomes through both paracellular and transcellular pathways, with paracellular transport assuming significance in pathological contexts, such as inflammation [116]. The transcellular pathway entails the endocytosis of exosomes by endothelial cells, followed by their transport across the cellular body for subsequent release on the opposite side [117]. Moreover, the lymphatic system is implicated in the transit of exosomes from various organs into the bloodstream, suggesting its critical role in the systemic dissemination of exosomes [114].

Endothelial cells are known to internalize exosomes through diverse mechanisms, including clathrin-dependent endocytosis, caveolin-mediated uptake, macropinocytosis, and lipid raft-mediated internalization [54]. These uptake pathways are influenced by the exosomes biophysical attributes, which may, in turn, modulate the endothelial internalization profile [117]. This inherent variability in exosome properties could be consequential in determining their cellular uptake dynamics, a phenomenon similarly observed with synthetic nanoparticles [54,117]. For instance, larger particles are predominantly internalized through phagocytosis or macropinocytosis, whereas smaller counterparts are primarily subject to alternative endocytic pathways [54,118]. This intricate interplay between exosomes and endothelial cells highlights the complexity of cellular internalization mechanisms and the potential for differential pathway engagement based on exosomal characteristics [114].

### 4.6. Functional Delivery of Exosomal Cargo

Cells encapsulate specific molecular cargoes within exosomes, which are then released into the extracellular milieu [88]. The lipid bilayer membrane of exosomes serves a critical protective role, shielding the encapsulated cargo from enzymatic degradation in the extracellular environment [1,119]. This process of paracrine signaling involves the direct transfer of this cargo into recipient cells, thereby exerting control over multiple levels, including genetic, signaling pathways, and overall cellular activities [97]. A key aspect of the therapeutic application of exosomes is ensuring the efficient release of the encapsulated content from the endosome before its internal environment becomes acidic and degrades the cargo [120]. However, challenges such as cargo degradation, intracellular recycling, or re-release of intact vesicles into the extracellular space can impede effective therapeutic delivery [12].

Once internalized by target cells, the exosomal cargoes are released, impacting various cellular processes based on the nature of the cargo and the physiological state of the donor cell [121]. For instance, mRNA and miRNA within exosomes can modify gene expression patterns in recipient cells, potentially leading to the synthesis of new proteins. Similarly, proteins and lipids carried by exosomes can also activate or inhibit specific signaling pathways [32], while antigens delivered via exosomes may modulate immune responses, enhancing or suppressing them as required. For example, cargos carried by exosomes activate the Wnt/β-catenin pathway for collagen deposition; the PI3K/AKT/mTOR pathway can activate endothelial cells or fibroblast functions, while the VEGF pathway promotes angiogenesis in wound healing processes [122]. 

The specificity with which exosomes target recipient cells offers a significant advantage in designing targeted therapeutic strategies, thereby increasing efficacy, and minimizing side effects [119]. This targeting capability also allows for the in vitro manipulation of exosomes to load them with specific therapeutic agents, including drugs such as curcumin, doxorubicin, paclitaxel [123], RNA interference molecules, or other bioactive compounds. Due to their natural origin and biocompatibility, exosomes generally evade immediate immune detection, which is particularly advantageous in drug delivery applications. For example, in oncological therapies, exosomes derived from immune cells can be engineered to carry tumor-suppressive agents such as miRNA, miR-199a-3p [124], cisplatin, doxorubicin [124,125] directly to cancer cells, thus inhibiting tumor growth and progression [126]. This nuanced understanding of exosomal functions and their potential applications underscores the transformative possibilities of exosomes in medical science, particularly in targeted and precision therapies [127].

## 5. Current Attempts to Overcome Challenges Associated with the Therapeutic Application of Exosomes

To address these concerns, a task force from the International Society for Extracellular Vesicles (ISEV) Rigor and Standardization Committee has outlined the pre-analytical factors essential for the research on blood-derived exosomes, aiming to enhance the reproducibility of exosome isolation from blood samples.

### 5.1. Standardization of Research

Efforts to standardize clinical research on exosomes have predominantly concentrated on the optimization of isolation and characterization protocols [53]. However, the preanalytical phases, encompassing specimen handling, storage, and collection protocols, substantially influence the reproducibility and integrity of research outcomes [128,129]. Among the critical preanalytical variables, the transportation duration of biological specimens, such as blood, wound exudates, urine, and other bodily fluids, exhibits significant variability in terms of temperature and transit time, even within identical specimen types [130]. This variability can markedly affect experimental results and should be meticulously considered in data interpretation, especially when samples originate from multiple collection sites. Explicit documentation of these preanalytical variables in the methods section is imperative to evaluate their impact on the research findings and to facilitate cross-study comparisons.

The present market offers a variety of commercial kits, such as exosome isolation kits by System Biosciences, Thermo Fischer Scientific, Qiagen, Miletnyi, Norgen Biotek, designed to isolate exosomes from specific biological sources [130]. These kits employ various methodologies, including polymer precipitation, membrane affinity, antibody capture, and filtration to separate or concentrate exosomes. These kits, while beneficial under certain conditions, must be used with caution, as they lack comprehensive documentation on the principles of exosome isolation and enrichment [130,131,132]. The absence of detailed methodological information can lead to the introduction of unknown impurities, such as polyethylene glycol, which can compromise the purity of the exosome preparations and potentially skew the experimental results. Such contaminants could mislead the interpretation of the data, affecting the validity of the research findings [37].

Therefore, it is recommended to select commercial exosome isolation kits that provide detailed procedural information for reproducibility. Standardizing these procedures not only enhances the reliability of the research but also supports the broader scientific community in achieving consistent and verifiable results in exosome-based studies.

### 5.2. Reporting Standards

The integrity of the data within Vesiclepedia (http://www.microvesicles.org/), derived from both curated and author-submitted publications, is directly linked to the quality of the corresponding EV research [133,134]. The field of exosomes is characterized by a lack of stringent nomenclature and variability in the exosome isolation protocols across different studies and laboratories, significantly affecting the purity and type of the isolated exosomes, as well as their associated cargoes [1]. Furthermore, it is imperative to note that the recurrent identification of certain proteins in exosome studies does not necessarily qualify them as definitive exosome markers or proteins enriched in exosomes. Consequently, users are advised to exercise caution when employing Vesiclepedia data for further analysis, paying particular attention to the isolation methods and meta-annotations to select high-quality datasets pertinent to their research [135]. As of the latest update, Vesiclepedia comprises data from 3533 EV-related studies, marking an over twofold increase in the database’s catalog since the 2019 update. This expansion includes contributions of 56,691 proteins, 50,550 RNA, 3839 lipids, 192 metabolites, and 167 DNA entries [133]. Quantitative data, now available for 62,822 entries derived from 47 studies, represent a significant augmentation of Vesiclepedia’s utility. The database presently catalogs 252 sample sources from 56 distinct organisms. The latest enhancement to Vesiclepedia introduces EVQUANT, a novel feature facilitating the relative quantification of extracellular vesicle (EV) proteins, RNA, and lipid cargoes within individual studies [133]. Given the diversity in experimental methodologies and sample processing across studies, it is currently not feasible to perform quantitative cross-comparisons. Nevertheless, the advent of high-throughput data generation and the potential for establishing uniform analytical pipelines herald the possibility of cross-study comparisons, contingent upon the standardization of experimental procedures. 

### 5.3. Accurately Defining the Vesicles

As per the MISEV 2023 guidelines [136], exosomes are defined as endosomal-derived intraluminal vesicles that form a specific subtype of small EVs with diameters less than 200 nm. This classification distinguishes them from other small EVs, such as ectosomes, which also form part of the broader EV population, but originate through different biogenetic processes. The terms “exosome” and “small EV” are not synonymous and should be used with precision. The lack of universal molecular markers for exosomes, ectosomes, and other EV subtypes presents a significant challenge in definitively characterizing these entities based on their origin. Consequently, much of the current research focuses broadly on mixed EV populations rather than exclusively on exosomes, pending clear evidence of their specific cellular origins [136].

### 5.4. Single-Exosome Studies

In the context of therapeutic applications, the characterization and validation of exosomes necessitate a comprehensive assessment, focusing on their size, shape, and molecular content [137]. Current methodologies for exosome characterization are broadly categorized into three primary domains: morphological analysis, size determination, and cargo profiling [138].

Morphological analysis employs techniques such as Scanning Electron Microscopy (SEM) and TEM, which facilitate the direct visualization of exosomal internal structures and surface topography, respectively [138]. Despite the detailed resolution offered by TEM, its intricate operation requirements [139] and labor-intensive sample preparation procedures limit its suitability for high-throughput analysis [140]. High-throughput analysis enables the simultaneous profiling of thousands of exosomes, facilitating the categorization of these vesicles based on their molecular signatures, such as lipids, proteins, and mRNA. This technology assists in identifying the origins and potential functions of exosomes [141]. Additionally, it can be employed to engineer exosomes and to screen them from various biological fluids, including blood, urine, and cerebrospinal fluid, enhancing their utility in disease diagnostics and prognostics [142]. This approach is instrumental in advancing our understanding of exosome-mediated processes and their implications in health and disease [143]. For size determination, methodologies such as nanoparticle tracking analysis (NTA), Dynamic Light Scattering (DLS), and tunable resistive pulse sensing (TRPS) are employed [144]. Among these, NTA stands out for its ability to provide high-resolution measurements, enabling the rapid identification and real-time observation of exosomes [145]. However, NTA’s capability to distinguish between exosomal particles and protein contaminants remains a challenge [146]. To overcome the limitations inherent in each characterization technique, it is a common practice to employ a multifaceted approach, integrating methods from each of the three domains, such as a combination of TEM, NTA, and protein-marker studies. This integrated strategy ensures a comprehensive characterization of exosomes, balancing the advantages and disadvantages of each method [137].

The diversity in biophysical and biochemical characteristics of exosomes is significantly influenced by the originating cell line and the employed isolation technique, leading to variability in the outcomes of biological analyses such as Western blots, PCR, and Dynamic Light Scattering (DLS) [147]. While traditional ensemble analysis methods have provided insights into the general biological and physical properties of exosomes, they offer limited resolution regarding the heterogeneity and individual characteristics of these vesicles. Consequently, these ensemble approaches, encompassing Western blot, enzyme-linked immunosorbent assay (ELISA), and Polymerase Chain Reaction (PCR), have been recognized for their limitations in accurately reflecting the complexity and diversity of exosomal populations [148].

In response to these limitations, advancements in analytical technologies have facilitated the shift towards single-particle analyses, enabling detailed investigation into the unique roles and properties of individual exosomes [149]. The guidelines proposed by the Minimal Information for Studies of Extracellular Vesicles (MISEV) advocate for the implementation of at least two single-particle analysis techniques to evaluate both the shape and biological attributes of single exosomes, emphasizing the importance of characterizing these vesicles at the individual level [150]. The development and refinement of single-exosome analysis techniques aim to address critical challenges associated with exosome research, including their intrinsic heterogeneity, measurement precision, complex biochemical composition, and nanoscale dimensions [11]. The increasing recognition of the need for precise sorting and phenotyping of specific exosome subpopulations has spurred the development of over twenty innovative single-vesicle methodologies [151]. Despite these advancements, the direct visualization of exosomes remains challenging due to their size, which often approaches the diffraction limit of standard optical microscopy. This limitation hampers the detailed examination of extracellular vesicle interactions and behaviors within cellular environments [152]. However, the advent of super-resolution imaging techniques, such as direct stochastic optical reconstruction microscopy (dSTORM), has opened new avenues for understanding the formation, function, and intracellular dynamics of extracellular vesicles [153]. dSTORM offers unprecedented sensitivity from free dyes or dye aggregations and resolution, enabling the precise determination of individual exosome arrangement, localization, and clustering through the tracking of vesicles in fluorescence mode with ultra-high single-molecule sensitivity [154,155,156]. Total internal reflection fluorescence (TIRF) microscopy, in synergy with single-molecule localization techniques, significantly enhances the signal-to-noise ratio and reduces the duration of imaging sessions [157]. However, super-resolution fluorescence microscopy demands sophisticated instrumentation and powerful data processing software, often resulting in low throughput [158]. While fluorescence microscopy techniques are pivotal for elucidating the mechanisms of exosome secretion, characterization, and uptake, the potential of fluorescent markers to interfere with their localization, activity, and functionality cannot be overlooked [150]. Additionally, lipid markers may exhibit non-specificity in their labeling properties or might aggregate [159]. 

In this context, flow cytometry (FCM) and nanoparticle tracking analysis (NTA) emerge as alternative approaches, leveraging the analysis of scattered light patterns from single particles and the tracking of light-scattering signals from particle diffusion, respectively, to derive physical insights at sub-wavelength scales [160]. Furthermore, fluorescence-based methodologies harness the interactions between light and molecules, employing fluorophore-conjugated target markers for labeling and detection [161]. The innovation of nano-flow cytometry (nFCM) by Prof. Yan Xiaomei’s laboratory represents a significant leap forward. By amalgamating Rayleigh light scattering with sheath flow single-molecule fluorescence detection technology, nFCM markedly enhances the sensitivity of both scattering and fluorescence detection relative to conventional FCM [162]. Capable of analyzing up to 10,000 particles per minute, this technology permits the multi-parametric quantitative measurement of single vesicles, down to a minimum particle size of 40 nm [150]. However, the advanced equipment and expertise required for nFCM impose limitations on its widespread clinical application. Furthermore, electron microscopy (EM) and atomic force microscopy (AFM) offer high-resolution imaging capabilities that allow for the direct visualization of exosomes, thus facilitating the characterization of their morphological and particle size attributes [163]. The application of these diverse nanotechnologies enables the comprehensive examination and cross-validation of the distinct traits and biological functions of individual extracellular vesicles (EVs) [164]. Consequently, researchers must judiciously select a combination of these techniques to effectively conduct their analyses. Therefore, the strategic integration of single-exosome analysis methodologies is a critical prerequisite for advancing our understanding of the physiological and pathological roles of exosomes [165]. This integrative approach not only elucidates the mechanisms underlying exosomal-mediated cell-to-cell communication but also paves the way for establishing a novel paradigm in exosome research [10,166].

### 5.5. Exosome Storage

Cryopreservation, lyophilization, and spray-drying are the predominant methodologies for the extended preservation of therapeutic exosomes, primarily relying on controlled temperature regulation and the inclusion of cryoprotective agents [167]. Although it is imperative to analyze exosomes in their native state post-isolation, for therapeutic purposes, extended shelf life is typically necessary. The stability of exosomes can vary, with some remaining intact without the need for freezing, depending on their biochemical composition and source of origin. Previous research has demonstrated that for long-term storage, a standard temperature of −80 °C is often used [168]. Specific studies have demonstrated that urinary exosomes can be preserved for up to four years at −20 °C, while saliva-derived exosomes may retain their protein composition and membrane integrity for up to 20 months when stored at 4 °C, although storage at this temperature can potentially diminish their protein content and biological activity [169].

The process of lyophilization offers an alternative storage solution, allowing exosomes to be easily preserved and reconstituted with the addition of an appropriate buffered solvent [170]. Recent studies suggest that lyophilization, particularly with the inclusion of cryoprotectants, can maintain the functionality of exosomal proteins and RNA for approximately four weeks, even at ambient temperatures [169]. The application of cryoprotectants is crucial in mitigating the adverse effects of freeze–thaw cycles, preventing cryodamage and aggregation of exosomes [171]. Formulations such as phosphate-buffered saline enriched with human albumin and the non-permeable disaccharide trehalose (PBS-HAT) have been found to enhance both short- and long-term stability and enhance both the short- and long-term exosome stability of therapeutic exosomes at −80 °C and across multiple freeze–thaw cycles [172,173]. The specifics of the freezing protocol (e.g., snap-freezing in liquid nitrogen vs. gradual freezing), the composition of the suspension buffer, storage duration, thawing techniques, and the number of freeze–thaw cycles are critical variables that need documentation, since optimal storage conditions can vary based on the exosomes’ origin and therapeutic composition [174]. To minimize detrimental freeze–thaw cycles, careful aliquoting is recommended, and it is crucial to recognize that samples subjected to varying numbers of freeze–thaw cycles may not be directly comparable. Proper labeling and documentation of storage containers are also essential to prevent the loss of exosomes due to adhesion to the container surfaces [174,175].

### 5.6. Modification of Exosomes for Specific Targeting

Exosomes, with their natural stability, low immunogenicity, and innate ability to target specific recipient cells represent an ideal vehicle for drug delivery [8]. These nanovesicles present a versatile platform for the bioengineering of therapeutic agents with minimal biochemical modification to enhance, broaden, or alter their therapeutic potentials [176]. The techniques for cargo loading into exosomes are categorized into pre- and post-biogenesis methodologies [177,178]. Pre-production strategies encompass methods such as transfection, co-incubation, and electroporation, implemented before the biogenesis of exosomes [179]. Post-production approaches include freeze–thaw cycles, incubation, sonication, extrusion, and hypotonic dialysis, applied after exosome formation [31,179,180]. For instance, Kim et al. utilized incubation and sonication to incorporate paclitaxel into exosomes derived from RAW 264.7 cells, aiming to counteract multidrug resistance in cancer therapy [181]. Similarly, Ohno et al. employed transfection to load anti-cancer let-7a miRNA into exosomes from HEK293 cells for breast cancer treatment [182].

The surface modification of exosomes represents another critical area of interest, achieved through the genetic manipulation of the exosomal membrane or the parental cells, the chemical conjugation of targeted ligands, electrostatic interactions, and the incorporation of magnetic nanoparticles [183]. The primary objective of these modifications is to achieve the targeted delivery of exosomes to specific cell types for precise therapeutic intervention [93]. Alvarez-Erviti et al. demonstrated targeted delivery to the central nervous system (CNS) by genetically engineering dendritic cells (DCs) to express Lamp2b fused with rabies viral glycoprotein (RVG) peptides [184]. In a similar vein, Zhu et al. chemically integrated c(RGDyK) tumor-targeting peptides onto exosomal surfaces to home in on glioblastoma cells [185]. 

Further innovations include the integration of α-EGFR, α-mCherry, and α-HER2 nanobodies onto the exosomal surface via phospholipid conjugation, effectively altering their targeting specificity in vitro [186]. This approach parallels strategies leveraging native exosomal membrane proteins, such as Lamp2b and platelet-derived growth factor receptors, as fusion partners for targeting ligands [186]. Additionally, chemical engineering methods such as click chemistry have been explored for surface modification, though the impact of these alterations on exosome–cell interaction dynamics and delivery efficacy remain under investigation [187]. Current research efforts are focused on elucidating the relationship between modified exosomal surface properties and their functional outcomes, with a view towards optimizing exosome-based therapeutic delivery systems [188,189].

Exosomes have pronounced membrane curvature as a consequence of their diminutive radius. This curvature arises through two principal mechanisms: first, ESCRT-mediated endosomal membrane deformation, leading to intraluminal vesicle budding within multivesicular endosomes [190]; second, neutral sphingomyelinase-induced ceramide production [191]. Based on these characteristics, an innovative approach to target lipid membranes effectively involves the design of peptides mimicking membrane-interacting proteins [82]. This approach could pioneer a new category of peptide sensors capable of simultaneous detection of phosphatidylserine (PS) and membrane curvature. Research findings indicate that the binding efficiency of the myristoylated alanine-rich C kinase substrate (MARCKS) effector domain (ED) is markedly reduced in the mutants MARCKSmut1 and MARCKSmut2, suggesting that MARCKS-ED can recognize PS-enriched, curved membranes [192]. This recognition is facilitated by phenylalanine (Phe) residues adapting to asymmetrically stretched bilayers and filling structural gaps in highly curved vesicles, thereby stabilizing membrane irregularities [193]. It was found that MARCKS-ED can differentiate between the sizes of lipid vesicles derived from both an animal model (rats) and synthetic phospholipid models, binding preferentially to highly curved membrane surfaces [193]. The effectiveness of curvature sensing is primarily dependent on the integration of aromatic Phe residues from the ED region into the lipid bilayers, as well as on the electrostatic interactions between the cationic residues (e.g., lysine, arginine) in the ED region and the negatively charged PS [194]. These lipid-targeting strategies offer new possibilities for probing exosomes in vivo or ex vivo, enhancing our ability to study critical biological phenomena such as apoptosis and vesicular shedding. Furthermore, MARCKS-ED-conjugated therapeutics could potentially achieve targeted delivery by binding to specific exosomes, followed by endocytosis, although the mechanisms of exosome cellular uptake remain under exploration [195]. 

In the context of infectious diseases, exosome-sized vesicles interconnecting red blood cells infected with malaria could enhance parasite survival under stress conditions [196]. MARCKS-ED targeting could disrupt intercellular communication via exosome-like vesicles between red blood cells infected with malaria, potentially delaying the development of drug resistance [197]. Advancing our understanding of exosomal functions in pathological conditions necessitates systematic peptide truncation to identify the minimal active sequence and essential residues for curvature detection by MARCKS-ED [194]. Enhancements in peptide affinity or specificity for specific exosomal populations could be attained through further residue modification and chemical optimization, including deletion, alanine scanning, and cyclization [198]. Investigating the membrane-binding capabilities of peptides derived from MARCKS-related proteins, which share nearly identical effector domains but differ by minor residue substitutions, could provide insights into how such modifications impact curvature sensing [194].

Further research involving systematic truncation is essential to determine the smallest active sequence and the minimal residues necessary for curvature sensing by MARCKS-ED [195]. Enhancements in binding affinity or specificity of MARCKS-derived peptides for cell-specific exosomes could be achieved through residue analysis and chemical optimization, including deletion, alanine scanning, and cyclization [199]. Additionally, evaluating the membrane-binding capabilities of peptide derivatives from a MARCKS homolog, which features a serine-to-proline substitution in the ED, could clarify whether this residue’s structural rigidity impacts curvature sensing [200]. An advanced method for visualizing the lipid composition and membrane curvature of exosomes would involve the application of bioorthogonal “click” chemistry to label the curvature probes with small molecule fluorophores, avoiding the use of large fluorescent proteins [201].

**Figure 3:**
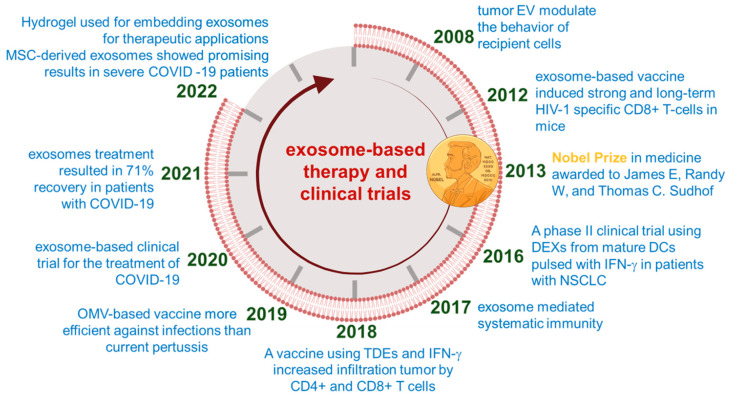
Timeline outlining the advancement in exosome-based therapy.

**Figure 4:**
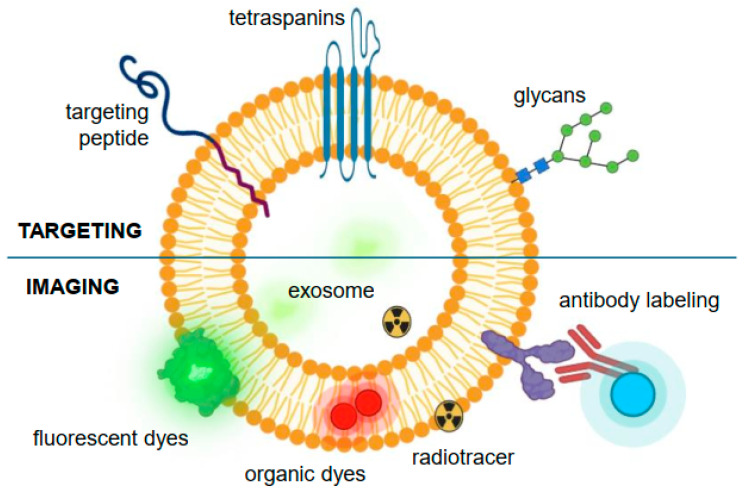
A schematic representation of the different strategies used for modifying the exosomal surface for targeted therapy and imaging applications. These strategies include labelling with antibodies, fluorescent dyes, radiolabeling, and other engineering techniques to enhance delivery specificity.

Elucidating the pharmacokinetic behavior of exosomes within biological systems and selecting an optimal delivery mechanism are paramount for determining the in vivo fate of these nanovesicles [202]. For the clinical advancement of exosome-based therapies (Figure 3) [26], it is imperative to consider several critical factors in the modification of exosome surfaces [203]. These include the selection of an appropriate targeting ligand or labeling agent, which requires a comprehensive understanding of the target site and the intended therapeutic application, all the while ensuring the absence of undesired immunogenic responses (Figure 4) [204]. Additionally, maintaining the physicochemical stability of exosomes necessitates the prevention of inadvertent alterations to their surface charge characteristics [176,204].

While covalent modification methods are frequently employed to affix labeling or targeting agents across the exosome surface, they present several challenges [205]. These techniques are limited to the attachment of genetically encodable proteins and peptides, excluding the possibility of targeting a broader range of molecules [206]. Moreover, the implementation of genetic modifications must not disrupt the intrinsic functions of proteins constituting the exosome membrane, given its complex nature [207].

The application of click chemistry for targeting specific proteins within the exosome membrane for modification is met with difficulties due to the membrane’s complexity [89]. However, advancements in the metabolic engineering of exosome parental cells have offered partial solutions to these challenges. This innovative approach involves the incorporation of artificially modified lipids, amino acids, and glycans to facilitate the expression of modified proteins on the exosome surface [208,209]. After this expression, additional chemical treatments are required to attach the desired labeling or targeting moieties, thus enabling the precision modification of exosomes for therapeutic purposes [209,210]. The utilization of click chemistry for exosome surface modification inherently poses a risk of chemical residue retention, which may elicit safety concerns regarding the modified exosomes and potentially complicate the regulatory approval process [211]. Additionally, the stability of aptamers under experimental conditions presents another significant challenge. Given these limitations, there is a growing preference for more efficient methods of exosome surface modification that circumvent these drawbacks [26,212].

An innovative approach emerging in this context involves the encapsulation of exosomes within phenol-metal-based nanofilms. This technique employs a coordination complex formed between tannic acid (TA) and Fe^3+^, onto which a targeting ligand can be functionalized via Michael addition to free amine groups [213]. For instance, this method has been applied to fabricate glutathione-capped gold nanoparticles by anchoring them to FA-functionalized, DOX-loaded exosomes (Exos-DOX-TA-Fe^3+^-FA), demonstrating the versatility and efficacy of this approach [213,214].

The TA-Fe^3+^ nanofilm offers commendable resistance against oxidants, heat, and ultraviolet (UV) light, presenting a robust platform for exosome modification [215]. However, it is crucial to assess the impact of such modifications on the structural integrity of the exosomes and the natural functions of their lipid, carbohydrate, and surface protein components [216]. Addressing these concerns meticulously is vital for harnessing the full potential of exosomes as an effective drug delivery system. Looking forward, the development of single-exosome analysis and the broader application of these novel technologies in a clinical setting will necessitate further clinical validation and the establishment of reproducibility [8]. This progression is essential for confirming the safety, efficacy, and reliability of exosome-based therapeutic delivery vehicles, paving the way for their successful integration into clinical practice [217].

## 6. Bio-Engineered Exosome Therapy in Precision Medicine

Exosome-based methodologies in precision medicine embody an integrative approach, combining molecular biology, nanotechnology, and individualized healthcare. They are poised to significantly enhance the prognosis, treatment, and monitoring of diseases in the future [218]. The foundational aspect of precision and personalized medicine involves the collection of patient-specific information, including lifestyle factors and genetic predispositions [219]. This information is crucial for devising tailored therapeutic strategies. Exosomes play a pivotal role in both the diagnosis of diseases and, potentially, preemptive therapeutic interventions, should early biomarkers be identified [220]. The advent of advanced “next generation” diagnostic techniques has facilitated the identification of myriad mutations and has enriched our understanding of the pathogenesis of various conditions, including cancers, neurological disorders, and infectious diseases [219,221]. These developments enable the formulation of individualized treatment regimens based on patient-specific biomarkers identified through exosomal analysis. A quantum leap in medical innovation is the concept of the “liquid biopsy,” a non-tissue-based biopsy method long-awaited for its minimal invasiveness [222,223]. This approach allows for the non-invasive screening, evaluation, monitoring, and diagnosis of diseases, garnering considerable interest due to its low-risk profile [219]. Recent advancements in liquid biopsy technologies have further streamlined the profiling of tumoral databases, encompassing methylation patterns and DNA/chromatin modifications [223]. Exosomes are integral to liquid biopsies, serving as carriers of biomarkers reflective of cellular conditions and being present across all body fluids [224]. The principal objective of precision medicine is the targeted treatment, or “ablation,” of pathological states with minimal collateral damage to healthy tissues, contingent upon the availability of comprehensive, personalized data sets [225]. Exosomes are particularly valuable in liquid biopsies for conditions such as pancreatic cancer, which are challenging to detect in their early, asymptomatic stages, and in circumstances where traditional tissue biopsies pose risks, such as during pregnancy [226].

Exosomes play a dual role in homeostatic processes; they can promote diseases, such as neuroinflammation and diabetes, and protect against them, facilitating immune homeostasis, tissue regeneration, and repair [227]. This dual functionality is pivotal as the utilization of exosomes as biomarkers and therapeutic agents in personalized medicine progresses [228]. Through interdisciplinary strategies, it is possible to load exosomes with specific cargoes, including DNA, RNA, oligonucleotides, proteins, and pharmaceuticals, drawing from extensive databases to maximize therapeutic efficacy. These modularized exosomes can be engineered with customizable surface molecules, enhancing their targeting capabilities, and minimizing damage to healthy tissues [224]. However, a significant challenge in the development of exosome-based personalized and precision medicine is the inherent heterogeneity of exosomes [8,229]. To address this complexity, a unified exosome profiling strategy that combines intradisciplinary expertise, advanced nanotechnology, and a multi-omics approach is essential. Additionally, comprehensive toxicological analysis is required to ensure the safety and effectiveness of exosome-based therapies. In this context, modularized exosomes, with their tailored compositions and targeting abilities, hold great promise for advancing the fields of precision and personalized medicine. 

The bioengineering of exosomes is an excellent approach to develop proper production standards and quality, but it may impair the outcomes by inaccurate targeting due to over-modification and leads to unintended side effects [230]. In addition, these manipulations could trigger adverse immune reactions, causing inflammation. Complex modifications could complicate the regulatory approval process, delaying clinical application [231]. Scaling up the production of bioengineered EVs, including exosomes, for large-scale applications presents several challenges and requires optimizations [29]. The isolation method includes ultracentrifugation, the density-gradient method, immunoprecipitation, which are labor-intensive and not easily scalable. By developing high-throughput methods for rapid and accurate characterizations and ensuring batch-to-batch consistency in surface modifications, it is possible to scale up the production of bio-engineered exosomes for clinical and commercial applications. Furthermore, there is a concern related to safety, which arises from the co-isolation of proteins, lipids, and other cellular debris, which can contaminate exosome preparations [230]. This can be overcome by implementing multi-step purification processes, such as combining size-exclusion chromatography with affinity capture, which can enhance purity. Additionally, there are risks associated with the high shear forces during isolation and processing, which can damage exosomes. Therefore, using gentle processing techniques such as tangential flow filtration can minimize the damage and maintain exosome integrity by reducing shear stress and efficiently separating exosomes from other vesicles [230].

## 7. Future Perspective and Conclusions

As the field of exosome research continues to evolve, the horizon for their application in therapeutic contexts appears increasingly promising. The future of exosome-based therapies is poised at the confluence of technological innovation, deeper biological understanding, and regulatory refinement. Key areas of focus will likely include the enhancement of exosome isolation and purification techniques to achieve higher yield and purity, which is critical for clinical applications. Furthermore, the development of scalable manufacturing processes that can maintain the functional integrity of exosomes will be essential to meet the demands of clinical trials and subsequent therapeutic use. Advancements in the genetic and biochemical surface modification of exosomes will enable more precise targeting and cargo delivery, opening new avenues for the treatment of a wide range of diseases, from cancer to neurodegenerative disorders. The integration of cutting-edge technologies, such as CRISPR-Cas9 for genome editing within exosomes, could further enhance their therapeutic potential. Moreover, the exploration of synthetic and biomimetic exosomes presents an intriguing frontier that may overcome some of the limitations associated with natural exosomal systems. Regulatory considerations will remain at the forefront, with a need for standardized protocols and benchmarks that can streamline the approval process for exosome-based therapies. Collaborative efforts between researchers, clinicians, and regulatory agencies will be crucial to establishing a framework that ensures safety and efficacy without stifling innovation. It is necessary to conduct a thorough and time-consuming safety examination. When evaluating the regulatory needs for exosome therapeutic uses, the purity of exosomes is crucial. For approval, the FDA and other international regulatory bodies look for safety, effectiveness, potency, and purity. For therapeutic application, a pure product, devoid of impurities such proteins, peptides, cell-free DNA, and other cell detritus, is necessary [55]. Large animals or primates are now being used by more researchers to examine the safety and effectiveness of exosome products. Human safety regarding exosomes is becoming more widely acknowledged. Although the FDA has not yet authorized any exosome products for therapeutic use [22,23], they have provided specific guidelines to sponsors on how to supply the necessary chemistry, manufacturing, and control (CMC) data to ensure the safety of their products [232]. Furthermore, biologics intended for the mitigation, treatment, cure, or prevention of illness must meet strict regulations, set out by the FDA and other international regulatory bodies. Exosomes must be isolated and purified with strict quality, purity, potency, and repeatability standards. More controls are needed for the exosome alterations that follow. Exosome standards are probably going to include both the contents and the cells from where they originated. There needs to be more standardization and review of exosome release criteria. The exosome-based product age is almost upon us, thanks to advancements in separation technology and our growing understanding of exosomes [233]. Furthermore, the integration of computational biology, machine learning, and artificial intelligence in exosome research holds the potential to unravel complex biological interactions, predict therapeutic outcomes, and optimize treatment regimens. This multidisciplinary approach will not only enhance our understanding of exosomal functions but also streamline the development of exosome-based diagnostics and therapeutics.

In summary, the exploration of exosomes as vehicles for therapeutic delivery embodies a significant leap toward the realization of precision medicine and pharmaceutical innovation. Exosome-based therapies from bench to bedside are fraught with challenges, but they are undeniably paved with significant potential [234]. The journey from conceptual understanding to clinical application of exosome-based therapies encapsulates the collaborative effort of researchers, clinicians, and regulatory authorities, underscored by the commitment to scientific rigor and patient safety. The future of exosome-based therapies is not without its uncertainties, yet the foundation laid by current research efforts provides a robust basis for optimism. Continued exploration and innovation within this domain hold the promise of revolutionizing the way we approach disease treatment, offering hope for patient-centric, precision medical solutions that could fundamentally alter the therapeutic landscape. As we stand on the cusp of this promising frontier, the path forward is complex and demands a multidisciplinary effort. The evolution of exosome research stands as a testament to the relentless pursuit of knowledge and the quest for therapeutic innovation. The collective efforts of the scientific community, clinicians, and regulatory bodies are crucial for navigating the complex regulatory landscape, ultimately facilitating the delivery of these innovative therapies to patients, and heralding new paradigms in medical treatment and patient care. As we peer into the future, exosomes may well be at the heart of the next wave of breakthroughs in pharmaceutical sciences and medicine, offering a beacon of hope for patients around the globe.

## Figures and Tables

**Table 1:** Advantages and disadvantages of exosome, microvesicle, and liposome-based therapies.

Vesicle Type	Advantages	Disadvantages
Exosomes	- Small and stable structures; have a high capacity for carrying and protecting therapeutic cargoes such as miRNAs, mRNAs, proteins, and drugs - Ability to cross biological barriers, including the blood–brain barrier - Ability to deliver therapeutics to difficult-to-reach tissues due to small size - Active packaging of cargo - Fast uptake - More targeting capacity - Lower toxicity	- Limited clinical trials - Scalability for clinical use - Need to standardize the isolation techniques - Difficult to perform modifications because of complex structure
Microvesicles	- Nonimmunogenic - Lower toxicity - Easily targetable - Can carry a more diverse array of cargo, including larger molecules and organelles - Passive packaging of cargo	- Need to standardize the isolation techniques - Potential safety concerns related to the heterogeneous nature of microvesicles - Limited clinical trials - Scalability for clinical use
Liposomes	- Diversity of lipid compositions - Scalable synthesis - Ability to load with desired cargo - Surface and cargo can be easily modified - Active clinical research	- Not easily targetable - Low cellular uptake and potential complement activation - Premature drug release - Higher toxicity

**Table 2:** List of exosome-based clinical trials (as of 10 May 2024, listed on clinicaltrials.gov).

NCT Number	Study Title	Conditions	Phases
NCT05218759	Exosomes Detection for the Prediction of the Efficacy and Adverse Reactions of Anlotinib in Patients with Advanced NSCLC	Non-Small-Cell Lung Cancer	NA
NCT02822131	Phosphate in Blood Pressure Regulation	Hypertension	NA
NCT02579460	Reflux-Induced Oxidative Stress in Barrett’s Esophagus: Response, Repair, and Epithelial-Mesenchymal-Transition	Barrett’s Esophagus|Gastroesophageal Reflux Disease	NA
NCT06072794	A Proof-of-Concept Study to Evaluate Exosomes from Human Mesenchymal Stem Cells in Women with Premature Ovarian Insufficiency (POI)	Premature Ovarian Insufficiency|Diminished Ovarian Reserve	Phase 1
NCT05658094	Exosome Effect on Prevention of Hair loss	Hair Loss|Alopecia	NA
NCT02649465	SGLT2 Inhibitor Versus Sulfonylurea on Type 2 Diabetes With NAFLD	Non-alcoholic Fatty Liver Disease	Phase 4
NCT03109873	Metformin Hydrochloride in Affecting Cytokines and Exosomes in Patients with Head and Neck Cancer	Larynx|Lip|Oral Cavity|Pharynx	Early Phase 1
NCT04184076	Randomized Controlled Trial of Time-Restricted Feeding (TRF) in Acute Ischemic Stroke Patients	Fasting	NA
NCT05490173	The Pilot Experimental Study of the Neuroprotective Effects of Exosomes in Extremely Low Birth Weight Infants	Premature Birth|Extreme Prematurity|Preterm Intraventricular Hemorrhage|Hypoxia-Ischemia, Cerebral|Neurodevelopmental Disorders	NA
NCT02138331	Effect of Microvesicles and Exosomes Therapy on Î²-cell Mass in Type I Diabetes Mellitus (T1DM)	Diabetes Mellitus Type 1	Phase 2|Phase 3
NCT04459182	Circulating Exosomes and Endothelial Dysfunction in Patients with Obstructive Sleep Apneas Hypopneas Syndrome	Endothelial Dysfunction	NA
NCT03608631	iExosomes in Treating Participants with Metastatic Pancreas Cancer with KrasG12D Mutation	KRAS NP_004976.2: p. G12D|Metastatic Pancreatic Adenocarcinoma Pancreatic Ductal Adenocarcinoma Stage IV Pancreatic Cancer AJCC v8	Phase 1
NCT04388982	the Safety and the Efficacy Evaluation of Allogenic Adipose MSC-Exos in Patients with Alzheimer’s Disease	Alzheimer’s Disease	Phase 1|Phase 2
NCT01104220	Role of Immune System in Obesity-related Inflammation and Cardiometabolic Risk	Non-alcoholic Fatty Liver Disease|Metabolic Syndrome|Metabolically Abnormal Obesity|Metabolically Normal Obesity|Obesity	NA
NCT05073627	The Effect of Dicloxacillin on Oral Absorption of Drugs	Healthy Volunteers|Drug–Drug Interaction	Phase 1
NCT04153539	Acute Health Effects of Traffic-Related Air Pollution Exposure	Cardiovascular System|Respiratory System	NA
NCT05871463	Effect of Mesenchymal Stem Cells-derived Exosomes in Decompensated Liver Cirrhosis	Decompensated Liver Cirrhosis	Phase 2
NCT05692635	Reducing the Incidence of Symptomatic Brain Metastases with MRI Surveillance	Brain Metastases|Non-Small-Cell Lung Cancer Stage III	Phase 2
NCT05163626	Combined Aerobic Exercise and Cognitive Training in Seniors at Increased Risk for Alzheimer’s Disease	Alzheimer’s Disease	NA
NCT04131166	Precision Nutrition and Metabolic Function	Obesity|Insulin Resistance	NA
NCT03217266	Navtemadlin and Radiation Therapy in Treating Patients with Soft Tissue Sarcoma	Resectable Soft Tissue Sarcoma|Soft Tissue Sarcoma	Phase 1
NCT03027726	Prevention of Diabetes in Overweight/Obese Preadolescent Children	Overweight Children with Type 2 Diabetes Risk	NA
NCT06259435	Modulating Energy Density in Time-Restricted Eating	Insulin Resistance|Body Weight	NA
NCT04134676	Therapeutic Potential of Stem Cell Conditioned Medium on Chronic Ulcer Wounds	Chronic Ulcer	Phase 1
NCT05777876	Early Identification of TRD and Construction and Clinical Validation of NTBS Precision Technology	Treatment-Resistant Depression	NA
NCT05563766	A Phase II Trial to Evaluate the Effect of Itraconazole on Pathologic Complete Response Rates in Resectable Esophageal Cancer	Esophageal Adenocarcinoma|Esophageal Squamous Cell Carcinoma|Gastroesophageal Junction Carcinoma	Phase 2
NCT04460963	Role of Adrenomedullin in Leukemic Endosteal/Vascular Niches	Acute Myeloid Leukemia	NA
NCT04258735	Genetic Characteristics of Metastatic Breast Cancer Patients	Metastatic Breast Cancer	NA
NCT03410030	Trial of Ascorbic Acid (AA) + Nanoparticle Paclitaxel Protein Bound + Cisplatin + Gemcitabine (AA NABPLAGEM)	Pancreatic Cancer|Pancreas Cancer|Pancreatic Adenocarcinoma Resectable|Pancreatic Ductal Adenocarcinoma|Pancreas Metastases	Phase 1|Phase 2
NCT04747574	Evaluation of the Safety of CD24-Exosomes in Patients With COVID-19 Infection	SARS-CoV-2	Phase 1
NCT05969717	Induced Pluripotent Stem Cell Derived Exosomes for the Treatment of Atopic Dermatitis	Atopic Dermatitis	Early Phase 1
NCT02653339	Effectiveness of Qufeng Shengshi Fang on Treatment of Allergic Rhinitis.	Rhinitis, Allergic, Perennial	NA
NCT04266639	Rheo-Erythrocrine Dysfunction as a Biomarker for RIC Treatment in Acute Ischemic Stroke	Stroke, Acute|Ischemic Stroke|Cerebrovascular Disorders|Central Nervous System Diseases	NA
NCT05354141	Extracellular Vesicle Treatment for Acute Respiratory Distress Syndrome (ARDS) (EXTINGUISH ARDS)	Acute Respiratory Distress Syndrome|ARDS	Phase 3
NCT05616234	Exercise-induced Changes in Exosomes	Exosomes|Connective Tissue|Exercise	NA
NCT06279039	Clinical Observation of Exosomes in Patients After Q-switched Laser Surgery	Exosome|Skin Regeneration|Laser	NA
NCT03230019	Two-Lumen Catheterization for Lung Wedge Resection	Thoracic Surgery, Video-Assisted	NA
NCT03392441	Insulin Deprivation on Brain Structure and Function in Humans with Type 1 Diabetes	Diabetes Mellitus, Type 1|Diabetes|Diabetes Complications	NA
NCT04483219	Tyrosine Kinase Inhibitor (TKI) + Anti-PD-1 Antibody in TKI-responded Microsatellite Stability/Proficient Mismatch Repair (MSS/pMMR) Metastatic Colorectal Adenocarcinoma.	MSS|pMMR|Metastatic Colorectal Adenocarcinoma	Phase 2
NCT05475418	Pilot Study of Human Adipose Tissue Derived Exosomes Promoting Wound Healing	Wounds and Injuries	NA
NCT06110130	Effect of Empagliflozin on Podocyte Specific Proteins in African American Veterans With NDKD	Chronic Kidney Diseases	Phase 4
NCT05413148	The Effect of Stem Cells and Stem Cell Exosomes on Visual Functions in Patients with Retinitis Pigmentosa	Retinitis Pigmentosa	Phase 2|Phase 3
NCT04213248	Effect of UMSCs Derived Exosomes on Dry Eye in Patients With cGVHD	Dry Eye Disease	Phase 1|Phase 2
NCT05402748	Safety and Efficacy of Injection of Human Placenta Mesenchymal Stem Cells Derived Exosomes for Treatment of Complex Anal Fistula	Fistula Perianal	Phase 1|Phase 2
NCT03985696	Exosomes and Immunotherapy in Non-Hodgkin B-cell Lymphomas	Lymphoma, B-cell, Aggressive Non-Hodgkin (B-NHL)	NA
NCT05166616	Minnelide and Osimertinib for the Treatment of Advanced EGFR Mutated Non-Small Cell Lung Cancer	Advanced Lung Non-Small-Cell Carcinoma|Locally Advanced Lung Non-Small-Cell Carcinoma|Stage III Lung Cancer AJCC v8|Stage IIIA Lung Cancer AJCC v8|Stage IIIB Lung Cancer AJCC v8|Stage IIIC Lung Cancer AJCC v8|Stage IV Lung Cancer AJCC v8|Stage IVA Lung Cancer AJCC v8|Stage IVB Lung Cancer AJCC v8|Unresectable Lung Non-Small-Cell Carcinoma	Phase 1
NCT04556916	Early Detection of Prostate Cancer	Prostate Cancer	NA
NCT05815524	Physical Activity in Patients with Parkinson’s Disease: A “Disease Modifying” Intervention?	Parkinson’s Disease	NA
NCT04998058	Autogenous Mesenchymal Stem Cell Culture-Derived Signaling Molecules as Enhancers of Bone Formation in Bone Grafting	Bone Loss, Osteoclastic|Bone Loss, Alveolar|Alveolar Bone Loss|Alveolar Bone Atrophy|Grafting Bone	Phase 1|Phase 2
NCT02892734	Ipilimumab and Nivolumab in Treating Patients with Recurrent Stage IV HER2 Negative Inflammatory Breast Cancer	HER2/Neu Negative|Recurrent Inflammatory Breast Carcinoma|Stage IV Breast Cancer|Stage IV Inflammatory Breast Carcinoma	Phase 2
NCT04493242	Extracellular Vesicle Infusion Treatment for COVID-19 Associated ARDS	COVID-19|ARDS	Phase 2
NCT05698524	A Study of Temodar with Abexinostat (PCI-24781) for Patients with Recurrent Glioma	Recurrent High-Grade Glioma|Anaplastic Astrocytoma|Anaplastic Oligodendroglioma|Glioblastoma|Gliosarcoma	Phase 1
NCT04798716	The Use of Exosomes for the Treatment of Acute Respiratory Distress Syndrome or Novel Coronavirus Pneumonia Caused by COVID-19	COVID-19|Novel Coronavirus Pneumonia|Acute Respiratory Distress Syndrome	Phase 1|Phase 2
NCT04939324	Molecular Profiling of Exosomes in Tumor-draining Vein of Early-staged Lung Cancer	Lung Cancer|Exosomes|Non-Small-Cell Lung Cancer	NA
NCT05326724	The Role of Acupuncture-induced Exosome in Treating Post-stroke Dementia	Exosome|Post-stroke Dementia|Acupuncture	NA
NCT05621109	PRE-Pregnancy Weight Loss and the Reducing Effect on Childhood Overweight—Copenhagen	Overweight and Obesity|Weight Loss|Pregnancy Related	NA
NCT04641585	Brugada Syndrome and Artificial Intelligence Applications to Diagnosis	Brugada Syndrome 1	NA
NCT05610332	Clinical Efficacy in Neoadjuvant Treatment of Locally Advanced Gastric Cancer with Different Immunotypes	Locally Advanced Gastric Cancer	Phase 3
NCT02869685	Consistency Analysis of PD-L1s in Advanced NSCLC Tissues and in Plasma Exosomes Before and After Radiotherapy	NSCLC	NA
NCT06361485	Safety and Feasibility of Umbilical Cord Wharton’s Jelly Allograft Injections for Lumbar Pain	Low Back Pain	Phase 1
NCT02928432	SWITCH: Study of the Prednisone to Dexamethasone Change in mCRPC Patients Treated with Abiraterone	Prostate Cancer	Phase 2
NCT04765137	Evaluate the Effect of Atorvastatin on Cerebrovascular Reactivity in MCI	Mild Cognitive Impairment	Phase 2
NCT03854032	Nivolumab and BMS986205 in Treating Patients with Stage II-IV Squamous Cell Cancer of the Head and Neck	Lip|Oral Cavity Squamous Cell Carcinoma|Pharynx|Larynx|Squamous Cell Carcinoma	Phase 2
NCT04585932	Androgen Deprivation Therapy and Apalutamide With or Without Radiation Therapy for the Treatment of Biochemically Recurrent Prostate Cancer, RESTART Study	Biochemically Recurrent Prostate Carcinoma|Metastatic Prostate Carcinoma|Oligometastatic Prostate Carcinoma|Stage IV Prostate Cancer AJCC v8|Stage IVA Prostate Cancer AJCC v8|Stage IVB Prostate Cancer AJCC v8	Phase 2
NCT05669144	Co-transplantation of Mesenchymal Stem Cell Derived Exosomes and Autologous Mitochondria for Patients Candidate for CABG Surgery	Myocardial Infarction|Myocardial Ischemia|Myocardial Stunning	Phase 1 |Phase 2
NCT04029740	Exosomal microRNAs as a Biomarker in Panic Disorder and in Response to CBT	Panic Disorder	NA
NCT06339840	The Impact of Lifestyle Intervention on Weight and Fertility in Obese Males	Obesity|Weight Loss|Male Fertility|Artificial Insemination|IVF-ET	NA
NCT05192694	Evaluation of Fapi-pet in Prostate Cancer.	Prostate Cancer	NA
NCT04781062	Development of a Horizontal Data Integration Classifier for Noninvasive Early Diagnosis of Breast Cancer	Breast Cancer	NA
NCT03762629	Exercise and Diet Restriction on Cardiovascular Function in Obese Children and Adolescents	Childhood Obesity|Adolescent Obesity	NA
NCT04849429	Intra-discal Injection of Platelet-rich Plasma (PRP) Enriched with Exosomes in Chronic Low Back Pain	Chronic Low Back Pain|Degenerative Disc Disease	Phase 1
NCT05927129	Research on the Biological Mechanism of the Efficacy of Psychotherapy for Depression Based on the fNIRS	Depressive Disorder	NA
NCT05738629	Safety and Efficacy of Pluripotent Stem Cell-derived Mesenchymal Stem Cell Exosome (PSC-MSC-Exo) Eye Drops Treatment for Dry Eye Diseases Post Refractive Surgery and Associated with Blepharospasm	Dry Eye Disease	Phase 1|Phase 2
NCT05798494	PRE-Pregnancy Weight Loss and the Reducing Effect on Childhood Overweight - Aarhus	Overweight and Obesity|Weight Loss|Pregnancy Related|Child Nutrition Sciences|Body Composition	NA
NCT05261360	Clinical Efficacy of Exosome in Degenerative Meniscal Injury	Knee; Injury, Meniscus (Lateral) (Medial)|Meniscus Tear|Meniscus Lesion|Meniscus; Degeneration|Meniscus; Laceration|Meniscus Injury, Tibial|Knee Injuries|Knee Pain Swelling|Arthralgia	Phase 2
NCT03406780	A Study of CAP-1002 in Ambulatory and Non-Ambulatory Patients with Duchenne Muscular Dystrophy	Muscular Dystrophies|Muscular Dystrophy, Duchenne|Muscular Disorders, Atrophic|Muscular Diseases|Neuromuscular diseases|Nervous System Diseases|Genetic Diseases, X-Linked|Genetic Diseases, Inborn	Phase 2
NCT04334603	Exercise Intervention in Heart Failure	Heart Failure	NA
NCT04595903	Treatment of SARS-CoV-2 Virus Disease (COVID-19) in Humans with HemopurifierÂ^®^ Device	COVID-19	NA
NCT03711890	Ultra-High Resolution Optical Coherence Tomography in Detecting Micrometer Sized Early-Stage Pancreatic Cancer in Participants With Pancreatic Cancer	Pancreatic Carcinoma|Pancreatic Intraductal Papillary Mucinous Neoplasm, Pancreatobiliary-Type	NA
NCT05955521	Exosome as the Prognostic and Predictive Biomarker in EBC Patients	Triple Negative Breast Cancer|HER2-positive Breast Cancer	NA
NCT03493984	Plant Exosomes and Patients Diagnosed with Polycystic Ovary Syndrome (PCOS) 17	Polycystic Ovary Syndrome	NA
NCT03260179	Study to Evaluate the Safety, Preliminary Efficacy and Pharmacokinetics of 3810	Advanced Solid Tumor|Advanced/Metastatic Colorectal Cancer	Phase 1
NCT05286684	Feasibility of Exosome Analysis in Cerebrospinal Fluid During the Diagnostic Workup of Metastatic Meningitis (Exo-LCR)	Breast Cancer	NA
NCT04173650	MSC EVs in Dystrophic Epidermolysis Bullosa	Dystrophic Epidermolysis Bullosa	Phase 1|Phase 2
NCT05243368	Evaluation of Personalized Nutritional Intervention on Wound Healing of Cutaneous Ulcers in Diabetics	Foot, Diabetics	NA
NCT04421872	The Disorder of Circadian Clock Gene and Early Cognitive Dysfunction After General Anesthesia	Postoperative Delirium|General Anesthesia|Exosomes|Clock Gene Circadian Rhythm Disorders	NA
NCT04969172	A Phase II Randomized, Double-blind, Placebo-controlled Study to Evaluate the Safety and Efficacy of Exosomes Overexpressing CD24 to Prevent Clinical Deterioration in Patients with Moderate or Severe COVID-19 Infection	COVID-19 Disease	Phase 2
NCT01294072	Study Investigating the Ability of Plant Exosomes to Deliver Curcumin to Normal and Colon Cancer Tissue	Colon Cancer	NA
NCT04879810	Plant Exosomes +/− Curcumin to Abrogate Symptoms of Inflammatory Bowel Disease	Irritable Bowel Disease	NA
NCT01550523	Pilot Immunotherapy Trial for Recurrent Malignant Gliomas	Malignant Glioma of Brain	Phase 1
NCT02439008	Early Biomarkers of Tumor Response in High Dose Hypo Fractionated Radiotherapy Word Package 3: Immune Response	Carcinoma, Hepatocellular|Colorectal Neoplasms|Melanoma|Kidney Neoplasms	NA
NCT03974204	Analyses of Exosomes in the Cerebrospinal Fluid for Breast Cancer Patients with Suspicion of Leptomeningeal Metastasis.	Breast Cancer|Leptomeningeal Metastasis	NA
NCT05913960	Accelerated Intermittent Theta-Burst Stimulation Ameliorate Major Depressive Disorder by Regulating CAMKII Pathway	Major Depressive Disorder|Major Depressive Disorder, Recurrent	NA
NCT05375604	A Study of exoASO-STAT6 (CDK-004) in Patients with Advanced Hepatocellular Carcinoma (HCC) and Patients with Liver Metastases from Either Primary Gastric Cancer or Colorectal Cancer (CRC)	Advanced Hepatocellular Carcinoma (HCC)|Gastric Cancer Metastatic to Liver|Colorectal Cancer Metastatic to Liver	Phase 1
NCT02706262	Complex Effects of Dietary Manipulation on Metabolic Function, Inflammation and Health	Obesity|Insulin Resistance	NA
NCT05216562	Efficacy and Safety of EXOSOME-MSC Therapy to Reduce Hyper-inflammation In Moderate COVID-19 Patients	SARS-CoV2 Infection	Phase 2|Phase 3
NCT05775146	SBRT of Metastases Following Neo-Adjuvant Treatment for Colorectal Cancer with Synchronous Liver Metastases	Colorectal Cancer|Liver Metastasis Colon Cancer	Phase 2
NCT02737267	Development of a Nutrigenetic Test for Personalized Prescription of Body Weight Loss Diets (Obekit)	Body Weight Changes	NA
NCT05524974	Clinical Study of Camrelizumab, Apatinib Mesylate and Nab-paclitaxel Combined with Oxplatin and S-1 in the Neoadjuvant Treatment of Locally Advanced Gastric Cancer With Different Genotypes	Locally Advanced Gastric Cancer	Phase 2
NCT06116903	Clinical Relevance of Detecting Molecular Abnormalities in Glial Tumor Exosomes	Glioma	NA
NCT04664738	PEP on a Skin Graft Donor Site Wound	Skin Graft	Phase 1
NCT04530890	Interest of Circulating Tumor DNA in Digestive and Gynecologic/Breast Cancer	Breast Cancer|Digestive Cancer|Gynecologic Cancer|Circulating Tumor DNA|Exosomes	NA
NCT02565264	Effect of Plasma Derived Exosomes on Cutaneous Wound Healing	Ulcer	Early Phase 1
NCT04542902	Non-coding RNAs Analysis of Eosinophil Subtypes in Asthma	Allergic Asthma|Severe Eosinophilic Asthma	NA
NCT02662621	Pilot Study with the Aim to Quantify a Stress Protein in the Blood and in the Urine for the Monitoring and Early Diagnosis of Malignant Solid Tumors	Cancer	NA
NCT05813379	Mesenchymal Stem Cells Derived Exosomes in Skin Rejuvenation	Anti-Aging	Phase 1|Phase 2
NCT03255408	Cerebral Blood Flow and Ventilatory Responses During Sleep in Normoxia and Intermittent Hypoxia	Obstructive Sleep Apnea of Adult|Hypoxia, Brain|Sleep Apnea|Sleep Disorder|Stroke|Blood Pressure|Endothelial Dysfunction|Oxidative Stress	Phase 1|Phase 2
NCT05043181	Exosome-based Nanoplatform for Ldlr mRNA Delivery in FH	Familial Hypercholesterolemia	Phase 1
NCT05110781	Atezolizumab Before Surgery for the Treatment of Regionally Metastatic Head and Neck Squamous Cell Cancer with an Unknown or Historic Primary Site	Locally Advanced Cutaneous Squamous Cell Carcinoma of the Head and Neck|Resectable Cutaneous Squamous-Cell Carcinoma of the Head and Neck|Stage III Cutaneous Squamous Cell Carcinoma of the Head and Neck AJCC v8	Phase 2
NCT05243381	Inflammation, NK Cells, Antisense Protein and Exosomes, and Correlation with Immune Response During HIV Infection	HIV Infections	NA
NCT05373381	The KetoGlioma (Ketogenic Glioma) Study	Glioma	NA
NCT03202212	Effect of Mixed On-line Hemodiafiltration on Circulating Markers of Inflammation and Vascular Dysfunction	Chronic Kidney Failure|Dialysis-Related Complication	Phase 1|Phase 2
NCT01811381	Curcumin and Yoga Therapy for Those at Risk for Alzheimer’s Disease	Mild Cognitive Impairment	Phase 2
NCT03459703	Effect of Time-Restricted Feeding on Fat Loss and Cardiometabolic Risk Factors in Overweight Adults	Obesity	NA
NCT05624203	Clinical Efficacy of Extracorporeal Cardiac Shock Wave Therapy in Patients with Ischemia-reperfusion Injury	Myocardial Reperfusion Injury|Treatment Outcome|Prognosis|ST Elevation Myocardial Infarction	NA
NCT04235023	PTP1B Implication in the Vascular Dysfunction Associated with Obstructive Sleep Apnea	Sleep Apnea|Inflammation|Atherosclerosis	NA
NCT05480150	Chinese Longitudinal and Systematic Study of Bioplar Disorder	Major Depressive Disorder|Bipolar Disorder, Mixed|Affective; Disorder, Organic	NA
NCT05832255	An Investigation of Psilocybin on Behavioural and Cognitive Symptoms of Adults with Fragile X Syndrome	Fragile X Syndrome|Behavior|Cognitive Dysfunction	Phase 2
NCT06245746	UCMSC-Exo for Chemotherapy-induced Myelosuppression in Acute Myeloid Leukemia	Acute Myeloid Leukemia|Neutropenia|Anemia|Thrombocytopenia|Infections|Bleeding	Phase 1
NCT04453046	Hemopurifier Plus Pembrolizumab in Head and Neck Cancer	Squamous Cell Carcinoma of the Head and Neck	NA
NCT04142138	When the Kidney Reacts to Nutritional Changes	Prehypertension	NA
NCT05833568	Five-day 20-min 10-Hz tACS in Patients with a Disorder of Consciousness	Brain Injury Traumatic Severe|Disorder of Consciousness|Brain Injuries	NA
NCT02977468	Effects of MK-3475 (Pembrolizumab) on the Breast Tumor Microenvironment in Triple Negative Breast Cancer	Triple Negative Breast Cancer	Phase 1
NCT06236568	Impact of Graft Reconditioning with Hypothermic Machine Perfusion on HCC Recurrence After Liver Transplantation	Hepatocellular Carcinoma|Liver Transplant; Complications	NA
NCT02748369	In vivo Assessment of Cellular Metabolism in Humans	Normal Cellular Metabolism	Phase 1
NCT04500769	Training Induced Muscle Exosome Release	Metabolism	NA
NCT02051101	Pathogenic Mechanisms of Port Wine Stain and Repository of Port Wine Stain Biopsy Samples	Port-Wine Stain	NA
NCT04602104	A Clinical Study of Mesenchymal Stem Cell Exosomes Nebulizer for the Treatment of ARDS	Acute Respiratory Distress Syndrome	Phase 1|Phase 2
NCT04491240	Evaluation of Safety and Efficiency of Method of Exosome Inhalation in SARS-CoV-2 Associated Pneumonia.	COVID-19|SARS-CoV-2 PNEUMONIA|COVID-19	Phase 1|Phase 2
NCT04653740	Omic Technologies to Track Resistance to Palbociclib in Metastatic Breast Cancer	Advanced Breast Cancer	NA
NCT03096340	Safety and Pharmacokinetic Study of IT-141 in Monotherapy in Patients with Advanced Cancer	Cancer|Neoplasms|Tumors|Refractory Solid Tumors|Recurrent Solid Tumors	Phase 1
NCT03478410	Role of Exosomes Derived from Epicardial Fat in Atrial Fibrillation	Atrial Fibrillation	NA
NCT06138210	The Effect of GD-iExo-003 in Acute Ischemic Stroke	Acute Ischemic Stroke	Phase 1
NCT05787288	A Clinical Study on Safety and Effectiveness of Mesenchymal Stem Cell Exosomes for the Treatment of COVID-19.	COVID-19 Pneumonia	Early Phase 1
NCT04965961	The Effect of Micro-doses Erytropoietin on Exercise Capacity in Male and Females	Sports Drug Abuse	NA
NCT03384433	Allogenic Mesenchymal Stem Cell Derived Exosome in Patients with Acute Ischemic Stroke	Cerebrovascular Disorders	Phase 1|Phase 2
NCT06319287	Phase 2a Multi-Center Prospective, Randomized Trial to Evaluate the Safety & Efficacy of Topical PEP-TISSEEL for Diabetic Foot Ulcers (DFU)	Diabetic Foot Ulcer	Phase 2
NCT04276987	A Pilot Clinical Study on Inhalation of Mesenchymal Stem Cells Exosomes Treating Severe Novel Coronavirus Pneumonia	Coronavirus	Phase 1
NCT06051461	Deciphering the Role of Dietary Fatty Acids on Extracellular Vesicles-mediated Intercellular Communication	Obesity|Metabolic Syndrome|Metabolic Disorder|Inflammation|Immune System and Related Disorders	NA
NCT06239207	Efficacy and Safety of Exosomes Versus Platelet Rich Plasma in Patients of Androgenetic Alopecia	Androgenetic Alopecia	Phase 2
NCT05933707	Small Extracellular Vesicles and Insulin Action	Obesity|Insulin Resistance|Metabolically Healthy Obesity|Obesity, Metabolically Benign	NA
NCT01159288	Trial of a Vaccination with Tumor Antigen-loaded Dendritic Cell-derived Exosomes	Non-Small-Cell Lung Cancer	Phase 2
NCT04536688	A Study of RGLS4326 in Patients with Autosomal Dominant Polycystic Kidney Disease	Polycystic Kidney Disease, Autosomal Dominant	Phase 1
NCT05060107	Intra-articular Injection of MSC-derived Exosomes in Knee Osteoarthritis (ExoOA-1)	Osteoarthritis, Knee	Phase 1
NCT04250493	Insulin Resistance in Multiple System Atrophy	Multiple System Atrophy	NA
NCT05523011	Safety and Tolerability Study of MSC Exosome Ointment	Psoriasis	Phase 1
NCT05669261	Treatment of Long COVID Symptoms Utilizing Autologous Stem Cells Following COVID-19 Infection	Long COVID	Phase 1
NCT05499156	Safety of Injection of Placental Mesenchymal Stem Cell Derived Exosomes for Treatment of Resistant Perianal Fistula in Crohn’s Patients	Perianal Fistula in Patients with Crohn’s Disease	Phase 1|Phase 2
NCT02507583	Antisense102: Pilot Immunotherapy for Newly Diagnosed Malignant Glioma	Malignant Glioma|Neoplasms	Phase 1
NCT03660683	Effect of Saxagliptin and Dapagliflozin on Endothelial Progenitor Cell in Patients with Type 2 Diabetes Mellitus	Diabetes Mellitus, Type 2|Cardiovascular diseases	Phase 4
NCT05318898	Effect of Dietary Protein on the Regulation of Exosome microRNA Expression in Patients with Insulin Resistance.	Insulin Resistance	NA
NCT06244849	Toward a Better Understanding of the Autophagy Machinery for the Identification of Potential Novel Biomarkers and Therapeutic Targets in Crohn’s Disease - TOPIC Study	Crohn’s Disease	NA
NCT03824275	18F-DCFPyL Positron Emission Tomography (PET)/Computed Tomography (CT) in Men with Prostate Cancer	Prostatic Neoplasms	Phase 2|Phase 3
NCT05864534	Phase 2a Immune Modulation with Ultrasound for Newly Diagnosed Glioblastoma	Newly Diagnosed Glioblastoma|Glioblastoma, Isocitric Dehydrogenase (IDH)-Wildtype|Gliosarcoma|Glioblastoma Multiforme	Phase 2
NCT04202770	Focused Ultrasound and Exosomes to Treat Depression, Anxiety, and Dementias	Refractory Depression|Anxiety disorders|neurodegenerative diseases	NA
NCT01668849	Edible Plant Exosome Ability to Prevent Oral Mucositis Associated with Chemoradiation Treatment of Head and Neck Cancer	Head and Neck Cancer|Oral Mucositis	Phase 1
NCT05843799	A Study to Evaluate the Safety and Tolerability of ILB-202	Healthy	Phase 1
NCT05228899	Zofin to Treat COVID-19 Long Haulers	COVID-19	Phase 1|Phase 2
NCT04270006	Evaluation of Adipose Derived Stem Cells Exo.in Treatment of Periodontitis	Periodontitis	Early Phase 1
NCT05259449	Efficacy of Educational Nutrition and Exercise on the Regulation of Appetite Through Exosomes in Type 2 Diabetics	Diabetes Mellitus, Type 2	NA
NCT02890849	Clinical Research for the Consistency Analysis of PD-L1 in Cancer Tissue and Plasma Exosome	NSCLC	NA
NCT02823613	The Influence of High and Low Salt on Exosomes in the Urine	Healthy	NA
NCT05871359	Transcranial Direct Current Stimulation and Dual Tasks	Parkinson’s Disease	NA
NCT03437759	MSC-Exos Promote Healing of MHs	Macular Holes	Early Phase 1
NCT03419000	Circulating microRNAs as Biomarkers of Respiratory Dysfunction in Patients with Refractory Epilepsy	Drug-Resistant Epilepsy	NA
NCT04427475	Prediction of Immunotherapeutic Effect of Advanced Non-small Cell Lung Cancer	NSCLC Patients	NA
NCT04602442	Safety and Efficiency of Method of Exosome Inhalation in COVID-19 Associated Pneumonia	COVID-19|SARS-CoV-2 PNEUMONIA|COVID-19	Phase 2
NCT03837470	Evaluation of Renal Sodium Excretion After Salt Loading in Heart Failure with Preserved Ejection Fraction	Heart Failure with Preserved Ejection Fraction	Early Phase 1
NCT03083678	Afatinib in Locally Advanced and Metastatic Chordoma	Chordoma	Phase 2
NCT05977088	Prediction of Effectiveness of rTMS Application in Alzheimer’s Patients	Alzheimer’s Disease	NA
NCT02706288	Effects of a Calorie Restricted, Very Low-Fat Plant-based Diet and Multi-component Exercise Program on Metabolic Health	Obesity|Insulin Resistance	NA
NCT06391307	The Role of Mesenchymal Stem Cell and Exosome in Treating Pilonidal Sinus Disease in Children	Pilonidal Sinus|Pilonidal Disease	NA
NCT04636788	Circulating Extracellular Exosomal Small RNA as Potential Biomarker for Human Pancreatic Cancer	Pancreas Adenocarcinoma	NA
NCT03700515	Exosome Proteomics to Detect EPO	Healthy	NA
NCT05387278	Safety and Effectiveness of Placental Derived Exosomes and Umbilical Cord Mesenchymal Stem Cells in Moderate to Severe Acute Respiratory Distress Syndrome (ARDS) Associated with the Novel Corona Virus Infection (COVID-19)	COVID-19 Acute Respiratory Distress Syndrome|Respiratory Distress Syndrome	Phase 1
NCT05834413	Clinical Study on the Prevention of Driver Gene Negative II-IIIa Lung Cancer Recurrence and Metastasis by Staged Chinese Herbal Medicine Combined With Chemotherapy and Immune Checkpoint Inhibitors	Lung Cancer	NA
NCT04389385	COVID-19 Specific T Cell Derived Exosomes (CSTC-Exo)	Corona Virus Infection|Pneumonia	Phase 1
NCT05808400	Safety and Efficacy of Umbilical Cord Mesenchymal Stem Cell Exosomes in Treating Chronic Cough After COVID-19	Long COVID-19 Syndrome	Early Phase 1
NCT04356300	Exosome of Mesenchymal Stem Cells for Multiple Organ Dysfunction Syndrome After Surgical Repair of Acute Type A Aortic Dissection	Multiple Organ Failure	NA
NCT03537599	Daratumumab and Donor Lymphocyte Infusion in Treating Participants with Relapsed Acute Myeloid Leukemia After Stem Cell Transplant	Minimal Residual Disease|Recurrent Acute Myeloid Leukemia with Myelodysplasia-Related Changes|Recurrent Adult Acute Myeloid Leukemia|Recurrent Childhood Acute Myeloid Leukemia	Phase 1|Phase 2
NCT04544215	A Clinical Study of Mesenchymal Progenitor Cell Exosomes Nebulizer for the Treatment of Pulmonary Infection	Drug-resistant	Phase 1|Phase 2
NCT03627611	Identification of Non-responders to Levothyroxine Therapy	Hypothyroidism|Biomarkers|Endocrine System Diseases	Phase 2
NCT04202783	The Use of Exosomes in Craniofacial Neuralgia	Neuralgia	NA
NCT05886205	Induced Pluripotent Stem Cell Derived Exosomes Nasal Drops for the Treatment of Refractory Focal Epilepsy	Refractory Focal Epilepsy	Early Phase 1
NCT04902183	Safety and Efficacy of Exosomes Overexpressing CD24 in Two Doses for Patients with Moderate or Severe COVID-19	COVID-19	Phase 2
NCT02535247	Study of MK-3475 Alone or in Combination with Copanlisib in Relapsed or Refractory NK and T-cell Non-Hodgkin Lymphoma	Lymphoma, T-Cell, Peripheral	Phase 1|Phase 2
NCT03228277	Olmutinib Trial in T790M (+) NSCLC Patients Detected by Liquid Biopsy Using BALF Extracellular Vesicular DNA	Non-Small-Cell Lung Cancer	Phase 2
NCT04313647	A Tolerance Clinical Study on Aerosol Inhalation of Mesenchymal Stem Cells Exosomes in Healthy Volunteers	Healthy	Phase 1
NCT04623671	Intravenous Infusion of CAP-1002 in Patients With COVID-19	COVID-19	Phase 2
NCT00285805	The Influence of Rosiglitazone on the Diuretic Effect of Furosemide and Amiloride	Insulin Resistance	NA
NCT04177498	Rigosertib in Patients with Recessive Dystrophic Epidermolysis Bullosa Associated SCC	Recessive Dystrophic Epidermolysis Bullosa	Early Phase 1
NCT01629498	Image-Guided, Intensity-Modulated Photon or Proton Beam Radiation Therapy in Treating Patients with Stage II-IIIB Non-small Cell Lung Cancer	Recurrent Lung Non-Small-Cell Carcinoma|Stage II Non-Small-Cell Lung Cancer AJCC v7|Stage IIA Non-Small-Cell Lung Carcinoma AJCC v7|Stage IIB Non-Small-Cell Lung Carcinoma AJCC v7|Stage IIIA Non-Small-Cell Lung Cancer AJCC v7|Stage IIIB Lung Non-Small-Cell Cancer AJCC v7	Phase 1|Phase 2
NCT03927898	Phase II Study of Toripalimab Plus Stereotactic Body Radiotherapy in Colorectal Cancer Patients with Oligometastasis	Metastatic Colorectal Cancer	Phase 2
NCT05947747	Safety and Efficacy of EXO-CD24 in Preventing Clinical Deterioration in Patients with Mild-Moderate ARDS	ARDS	Phase 2
NCT02594345	Effect of Exosomes Derived from Red Blood Cell Units on Platelet Function and Blood Coagulation	Blood Coagulation|Platelet Function	NA
NCT04384445	Zofin (Organicell Flow) for Patients With COVID-19	Corona Virus Infection|COVID-19|SARS|Acute Respiratory Distress Syndrome	Phase 1|Phase2
NCT04298398	Impact of Group Psychological Interventions on Extracellular Vesicles in People Who Had Cancer	Cancer	NA
NCT03109847	Metformin Hydrochloride in Mitigating Side Effects of Radioactive Iodine Treatment in Patients with Differentiated Thyroid Cancer	Thyroid	Phase 2
NCT04350177	A Study to Assess Single and Multiple Doses of IkT-148009 in Healthy Elderly Participants and Parkinson’s Patients	Healthy Elderly|Parkinson’s Disease	Phase 1
NCT05559177	An Open, Dose-escalation Clinical Study of Chimeric Exosomal Tumor Vaccines for Recurrent or Metastatic Bladder Cancer	Recurrent or Metastatic Bladder Cancer	Early Phase 1
NCT05607654	The Mechanisms of Underlying Accelerated Repetitive Transcranial Magnetic Stimulation for Treatment-resistant Depression	Treatment-Resistant Depression	NA
NCT06221787	Stem Cell Derived Exosomes in the Treatment of Melasma and Its Percutaneous Penetration	Melasma	NA
NCT02921854	Detection of Circulating Biomarkers of Immunogenic Cell Death	Non-Small-Cell Lung Cancer	NA
NCT02310451	Study of Molecular Mechanisms Implicated in the Pathogenesis of Melanoma. Role of Exosomes	Metastatic Melanoma	NA

## Abbreviations

AFM	atomic force microscopy
BMP-2	bone morphogenetic protein 2
CMC	chemistry, manufacturing, and control
CNS	central nervous system
DNA	deoxyribonucleic acid
dSTORM	direct stochastic optical reconstruction microscopy
DCs	dendritic cells
DLS	dynamic light scattering
ED	effector domain
EM	electron microscopy
ELISA	enzyme-linked immunosorbent assay
ECM	extracellular matrix
EV	extracellular vesicle
FCM	flow cytometry
FDA	food and drug administration
GMP	good manufacturing practice
HDL	high density lipoproteins
HIF-1α	hypoxia-inducible factor 1-alpha
IFN-γ	interferon-gamma
ISEV	international society for extracellular vesicles
IND	investigational new drug
IDL	intermediate-density lipoproteins
LDL	low density lipoproteins
LPS	lipopolysaccharide
miRNA	microRNA
MISEV	minimal information for studies of extracellular vesicles
MARCKS	myristoylated alanine-rich C kinase substrate
NTA	nanoparticle tracking analysis
nFCM	nano-flow cytometry
PCR	polymerase chain reaction
PBS-HAT	phosphate buffered saline-human albumin and trehalose
PS	phosphatidylserine
Phe	phenylalanine
qPCR	quantitative polymerase chain reaction
RNA	ribonucleic acid
RVG	rabies viral glycoprotein
SEM	scanning electron microscopy
TNF-α	tumor necrosis factor-alpha
TEM	transmission electron microscopy
TRPS	tunable resistive pulse sensing
TIRF	total internal reflection fluorescence
TA	tannic acid
UV	ultraviolet light
VLDL	very low-density lipoproteins
